# Mitochondrial dysfunction in some triple-negative breast cancer cell lines: role of mTOR pathway and therapeutic potential

**DOI:** 10.1186/s13058-014-0434-6

**Published:** 2014-09-11

**Authors:** Hélène Pelicano, Wan Zhang, Jinyun Liu, Naima Hammoudi, Jiale Dai, Rui-Hua Xu, Lajos Pusztai, Peng Huang

**Affiliations:** 1 Department of Translational Molecular Pathology, The University of Texas M.D. Anderson Cancer Center, 1515 Holcombe Boulevard, Houston, 77030 TX USA; 2 The First Affiliated Hospital of Nanchang University, Nanchang, Jiangxi China; 3 State Key Lab of Oncology in South China, Sun Yat-sen University Cancer Center, Guangzhou, China; 4 Department of Breast Medical Oncology, The University of Texas M.D. Anderson Cancer Center, Houston, 77030 TX USA; 5 Yale School of Medicine, New Haven, CT USA

## Abstract

**Introduction:**

Triple-negative breast cancer (TNBC) is a subtype of highly malignant breast cancer with poor prognosis. TNBC is not amenable to endocrine therapy and often exhibit resistance to current chemotherapeutic agents, therefore, further understanding of the biological properties of these cancer cells and development of effective therapeutic approaches are urgently needed.

**Methods:**

We first investigated the metabolic alterations in TNBC cells in comparison with other subtypes of breast cancer cells using molecular and metabolic analyses. We further demonstrated that targeting these alterations using specific inhibitors and siRNA approach could render TNBC cells more sensitive to cell death compared to other breast cancer subtypes.

**Results:**

We found that TNBC cells compared to estrogen receptor (ER) positive cells possess special metabolic characteristics manifested by high glucose uptake, increased lactate production, and low mitochondrial respiration which is correlated with attenuation of mTOR pathway and decreased expression of p70S6K. Re-expression of p70S6K in TNBC cells reverses their glycolytic phenotype to an active oxidative phosphorylation (OXPHOS) state, while knockdown of p70S6K in ER positive cells leads to suppression of mitochondrial OXPHOS. Furthermore, lower OXPHOS activity in TNBC cells renders them highly dependent on glycolysis and the inhibition of glycolysis is highly effective in targeting TNBC cells despite their resistance to other anticancer agents.

**Conclusions:**

Our study shows that TNBC cells have profound metabolic alterations characterized by decreased mitochondrial respiration and increased glycolysis. Due to their impaired mitochondrial function, TNBC cells are highly sensitive to glycolytic inhibition, suggesting that such metabolic intervention may be an effective therapeutic strategy for this subtype of breast cancer cells.

**Electronic supplementary material:**

The online version of this article (doi:10.1186/s13058-014-0434-6) contains supplementary material, which is available to authorized users.

## Introduction

Breast cancer is the most common malignant tumor in women and is a heterogeneous disease that exhibits various biological characteristics and clinical behaviors. Clinical subtypes of breast cancers are defined based on the presence or absence of estrogen receptors (ER), progesterone receptors (PR), and human epidermal growth factor receptor-2 (HER2). The majority (>60%) of breast cancers are ER-positive [1], whereas about 20% are negative for ER, PR, and HER2 expression (that is triple-negative breast cancer, TNBC) and most of these cancers have unfavorable clinical prognosis [2]. Despite significant improvements in breast cancer diagnosis and treatment, TNBC remains incurable using currently available drugs. Developing new therapeutic strategies and novel compounds effective in killing TNBC cells are urgently needed to improve the treatment outcome of TNBC patients.

Because TNBC cells lack specific cell-surface receptors for therapeutic targeting, one potential strategy to effectively kill these malignant cells would be to impact their unique metabolic properties. Cancer cells are more active in glycolysis (even in the presence of oxygen) to generate ATP and other metabolic intermediates for cell proliferation. This metabolic feature is known as the Warburg effect and is considered as a hallmark of cancer cells [3]-[6]. Although the mechanisms that alter the bioenergetic metabolism in cancer cells are still not fully understood, it is generally postulated that increased glycolysis offers cancer cells an advantage to better proliferate, survive and become invasive in the tumor microenvironment [4],[7]-[10]. The activities of hexokinase (HK), aldolase (ALD), pyruvate kinase (PK) and lactate dehydrogenase (LDH) are 3 to 7 times higher in human breast cancer than in normal tissue [11]-[13], although it is unclear if TNBC cells are particularly more active in glycolysis and more dependent on this pathway for ATP generation compared to other breast cancer cells. Furthermore, certain cancer cells may also actively use oxidative phosphorylation (OXPHOS) or a combination of OXPHOS and glycolysis for ATP production [13]-[16]. Thus, understanding the relative contribution of each pathway in different types breast cancer cells will enable us to identify if there is difference between TNBC cells and non-TNBC breast cancer cells and to design potential metabolic intervention strategies to effectively target TNBC cells.

The PI3K/AKT/mammalian target of rapamycin (mTOR) pathway plays a pivotal role in cell growth, proliferation and survival [17]. An overactive PI3K/AKT/mTOR pathway can be caused either by loss of tumor suppressor gene function (phosphatase and tensin homolog (PTEN), p53) or gain of PI3K function, leading to an increase in glucose uptake, glycolytic flux, and a switch from mitochondrial respiration to lactate production [18]-[20]. Recent reports have demonstrated that the PI3K/AKT/mTOR pathway is frequently altered in human breast cancer [21],[22] and that TNBC cells exhibit alterations in PTEN or loss in INPP4B [23]-[25]. However breast cancer cells harboring *PIK3CA* mutations are selectively sensitive to mTOR allosteric and kinase inhibitors but not in breast cancer cells with loss of PTEN, suggesting that the functional consequences of these two mechanisms of mTOR activation are quite distinct [26].

In this study, we investigated the metabolic alterations in TNBC cells in comparison with other subtypes of breast cancer cells, using molecular and metabolic analyses. We found that TNBC cells exhibited a significant decrease in oxygen consumption and a substantial increase in glucose uptake and lactate production compared to the receptor-positive cells. We further showed the mTOR pathway to be important in regulating OXPHOS in breast cancer cells and found that manipulation of expression of the key molecules such as p70S6K could significantly alter mitochondrial respiration and glucose metabolism. Furthermore, we showed that inhibition of glycolysis is highly effective in killing TNBC cells despite their resistance to other anticancer agents. These novel findings provide new insights into the metabolic biology of breast cancer, and may serve as a biochemical basis for developing effective therapeutics for TNBC cancers by selectively targeting their unique energy metabolism.

## Methods

### Cell cultures

Cells (BT474, MCF7, BT20, T47D, ZR751, SKBR3, MDA231, MDA468, MDA436) were obtained from the American Type Culture Collection (Manassas, VA, USA). Cells were cultured in DMEM-F12 medium (Cellgro, Mediatech Inc, Herndon, VA, USA) supplemented with 10% fetal bovine serum and 2 mM glutamine at 37°C, 5% CO_2_, and 95% humidity. The experiments performed in this study do not required Institute Ethics Board approval because only commercially available cell lines were used.

### Reagents

N,N,N’,N’-tetramethyl-p-phenylenediamine dihydrochloride (TMPD), β-actin antibody, oligomycin, rotenone, RU486, ascorbate, succinic acid, digitonin, cyanide 3-chlorophenylhydrazone (CCCP), antimycin A and 4-hydroxytamoxifen (4-HOT) were acquired from Sigma (St Louis, MO, USA). Rapamycin was from AG Scientific, Inc (San Diego, CA, USA); (^3^H) 2-deoxyglucose was acquired from Amersham Pharmacia Biotech (Piscataway, NJ, USA). Phospho-Akt, Phospho-AMPK, total AMPK, Glut-4, hexokinase I, GPX-1, p70S6K (total and phosphorylated form) and α/β tubulin antibodies were from Cell Signaling Technology, Inc (Danvers, MA, USA). Cytochrome *c*, Akt total, SCO2, Glut-1, hexokinase II antibodies were purchased from Santa Cruz Biotechnology, Inc (Santa Cruz, CA, USA). MitoProfile® Total OXPHOS Human WB Antibody Cocktail was obtained from Mitosciences (Eugene, OR, USA); 7-AAD, MitoSOX™ Red, MitoTracker™ Green and rhodamine-123 were purchased from Invitrogen/Molecular Probes (Carlsbad, CA, USA). p70S6K and MigR1 plasmids were from Addgene (Cambridge, MA, USA) and shRNA P70S6K were from Thermo Scientific/Dharmacon (Lafayette, CO, USA) and were transfected to the cells according to the method provided by the companies.

### Measurement of respiration activity, glucose uptake, lactate production and ATP level

Oxygen consumption, cellular glucose uptake, lactate production and ATP content were measured as an indication of mitochondrial dysfunction, as previously described [8],[27]. To analyze the mitochondrial respiratory complex activity in breast cancer cells with different receptor status we used the oxytherm system equipped with the Clark electrode. Briefly, basal oxygen consumption was first determined in intact cells (1 × 10^6 cells) resuspended in respiratory buffer containing 20 mM HEPES pH 7.4, 10 mM MgCl2 and 250 mM sucrose, followed by addition of rotenone (500 nM, complex I inhibitor), at 6 minutes, digitonin (15 mg/ml) at 9 minutes, succinate (10 mM, complex II substrate) at 13 minutes, antimycin A (5 mM, complex III inhibitor) at 19 minutes, and TMPD + ascorbic acid (0.5 mM and 2 mM respectively, complex IV substrates) at 22 minutes. In the presence of rotenone and succinate (digitonin was also added to make the biological membranes permeable to succinate), the oxygen consumption rate would represent the maximal activity of complex II-> CoQ- > III-represent the maximal activity of complex> IV. In the presence of antimycin A and ascorbic acid/TMPD, the oxygen consumption rate would represent the uncoupled respiration through complex IV. The overall oxygen consumption at complex IV reflects the combined electron transport activities from both complex I- > CoQ- > III- > IV and complex II- > CoQ- > III- > IV.

### Real-time metabolic analysis

Simultaneous multiparameter metabolic analysis of cell populations in culture was performed in the Seahorse XF24 extracellular flux analyzer (Seahorse Bioscience, Billerica, MA, USA) as described previously [28]. Briefly, breast cancer cells were seeded on XF24 V7 multi-well plates (50,000 cells per well for TNBC cells and 20,000 cells per well for ER-positive cells) and were pre-incubated overnight at 37°C in 5% CO_2_. The following day, the culture medium was replaced with assay media (unbuffered DMEM/F12 supplemented with 2 mM glutamine, pH 7.4) 1 hour before the assay and for the duration of the experiment. Mitochondrial complex inhibitors (10 ng/ml oligomycin, 4 μM CCCP, 1 μM rotenone) are preloaded in the injection ports. After establishing the baseline oxygen consumption rate (OCR) and extracellular acidification rate (ECAR) readings, mitochondrial complex inhibitors (oligomycin, CCCP, rotenone) were injected consequently and after a short period of mixing, oxygen consumption rate OCR and ECAR measurements were made using photodetectors with specific excitation and emission wavelengths of oxygen (532/650 nm) and protons (470/530 nm; 28). Experiments were run in quadruplicate. The data were normalized by cell number.

### Flow cytometry analysis of reactive oxygen species (ROS), mitochondrial mass, mitochondrial transmembrane potential (Δψm) and cell death

Cells were stained 60 minutes with 60 nM MitoTracker Green to measure the mitochondrial mass, with 3 μM Mitosox Red or 100 ng/ml dihydroethidium (DHE) to detect superoxide, or with 1 μM rhodamine-123 to evaluate the mitochondrial transmembrane potential, and with annexin-V-FITC (BD Biosciences Pharmingen, San Diego, CA, USA) and PI to analysis cell death according to the manufacturer’s protocol. Analysis was performed using a FACScan flow cytometer (Becton Dickinson Bioscience, San Jose, CA, USA) with the CellQuest software.

### In vitro proliferation assay

To quantify cell proliferation, we used the (3-(4,5-dimethylthiazol-2-yl)-5-(3-carboxymethoxyphenyl)-2-(4-sulfophenyl)-2H-tetrazolium, inner salt) (MTS) assay according to the manufacturer’s protocol (Promega Corporation, Madison, WI, USA). Each independent experiment was carried out three times.

### Analysis of cellular glutathione, NADH/NAD+, and NADPH/NAPD+

A glutathione assay kit (Cayman Chemical, Ann Arbor, MI, USA) was used to measure cellular glutathione; NADH/NAD + and NADPH/NADP + quantification kits (BioVision, Mountain View, CA) were used to determine NADH/NAD + and NADPH/NADP + ratios according to the manufacturer’s protocols.

### RNA isolation and reverse-transcription polymerase chain reaction (RT-PCR) analyses

RNA was isolated from breast cancer cells using the RNeasy kit and reverse-transcribed using the Omniscript RT kit (Qiagen, Hilden, Germany) according to the manufacturer’s instructions. The amplification primers for p70S6K were 5′-GTCGACAGCCCAGATGACTCAACTCTC-3′ (forward) and 5′-TCATAGATTCATACGCAGGTGCTCTGGC-3′ (reverse), for PDK1 were 5′- CCGCTCTCCATGAAGCAGTT- 3′ (forward) and 5′- TTGCCGCAGAAACATAAATGAG-3′ (reverse) and for β-actin were 5′-GCATCGTCACCAACTGGGAC-3′ (forward) and 5′-ACCTGGCCGTCAGGCAGCTC-3′ (reverse). cDNA was amplified using standard PCR conditions.

### Quantification

The quantification of western blots and PCR was performed using Image J software and can be found in Additional file 1.

### Statistical analysis

The *t*-test was used to evaluate the statistical differences of the experimental values between two samples to be compared.

## Results

### Bioenergetic profiles and redox status of TNBC cells in comparison with other breast cancer cells

**Figure 1:**
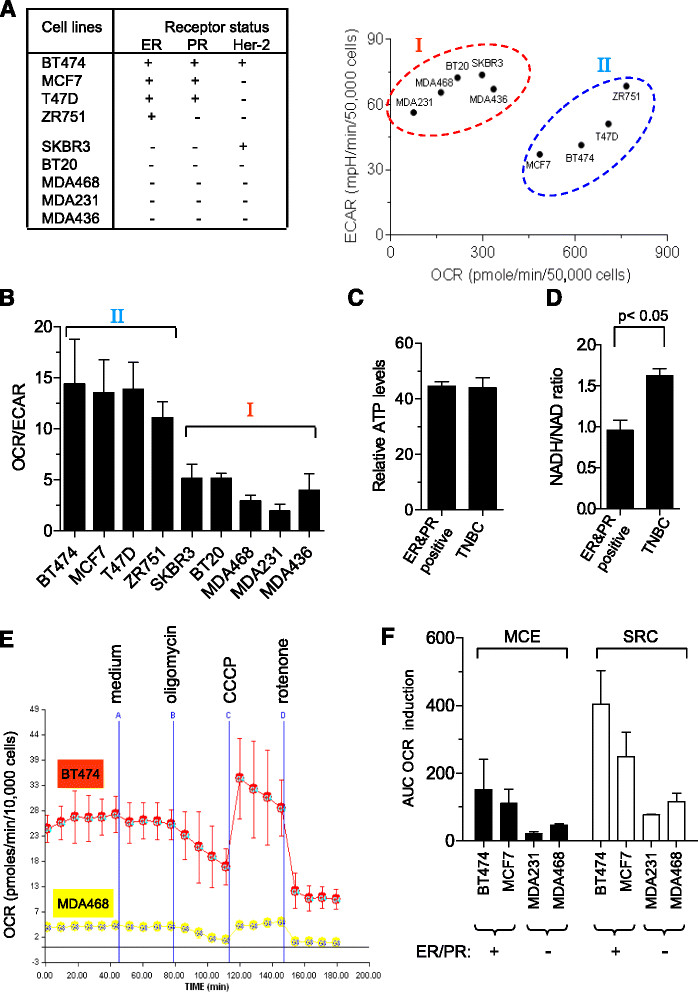
**Bioenergetic profiles of breast cancer cells with different expression of estrogen receptor (ER), progesterone (PR), and human epidermal growth factor-2 (HER2) using the XF24 analyzer. (A)** Breast cancer cell-receptor status used in this study (left) and breast cancer cell lines with different expression of ER, PR, and HER2 and their respective oxygen consumption rates (OCR) and acidification rates (ECAR) (right). Group I (BT20, MDA468, MDA231, MDA436, SKBR3) was compared to ER/PR-positive cells or Group II (BT474, MCF7, T47D, ZR751) (50,000 cells/well for each cell line). Results are represented as average fold-change ± SD of at least three independent experiments. **(B)** Ratio of OCR/ECAR in breast cancer cells with different expression of ER, PR, and HER2. **(C)** Comparison of ATP levels in breast cancer cells between the triple-negative breast cancer (TNBC) group (BT20, MDA468, MDA231, MDA436) and ER/PR-positive group (BT474, MCF7, T47D, ZR751). **(D)** Comparison of NADH/NAD^+^ ratios in breast cancer cell lines between the TNBC (BT20, MDA468, MDA231, MDA436) and ER/PR-positive group (BT474, MCF7, T47D, ZR751). **(E)** Comparison of mitochondrial respiratory function in BT474 (triple-positive) and MDA468 (triple-negative) breast cancer cells. Oxygen consumption in BT474 (20, 000 cells/well) and MDA468 cells (50,000 cells/well) was analyzed using XF24 under basal conditions (cellular assay medium), following by sequential additions of oligomycin (10 ng/ml), CCCP (4 μM), and then rotenone (1 μM) as indicated. OCR and ECAR were normalized by cell number in each well at the end of the experiments. For optimal analysis, we used 50,000 for TNBC cells (MDA486 and MDA231) and 20,000 for ER-positive cells (BT474 and MVF7) in this assay. **(F)** MCE and SRC in TNBC cells (MDA231, MDA468) in comparison with that of breast cancer cells expressing ER and PR (MCF7, BT474). The bar graph represents mean ± SD from three experiments.

We first evaluated the mitochondrial bioenergetic profiles of TNBC cells by measuring their oxygen consumption and glycolysis rates in comparison with other types of breast cancer cells. The nine cell lines used in this study and their ER, PR, and HER2 expression status are shown in Figure 1A (left panel). A Seahorse extracellular flux analyzer XF24 was used to measure the ECAR and OCR of each cell line, as the indicators of lactic acid production during glycolysis and mitochondrial respiration during OXPHOS, respectively. As shown in Figure 1A (right panel), all TNBC cell lines and the ER/PR double-negative, HER2-positive SKBR3 cells (group I) exhibited higher ECAR and lower OCR compared with the receptor-positive breast cancer cells (group II). Quantitative analysis revealed that TNBC cells had lower OXPHOS and higher glycolysis, as they exhibited significantly lower OCR/ECAR ratios than other breast cancer cells (Figure 1B). Using traditional biochemical analyses, we further validated that TNBC cells exhibited lower mitochondrial respiration and a compensatory increase in glycolysis, as evidenced by a decrease in oxygen consumption and an increase in glucose uptake and lactate production (Additional file 2: Figure S1A-C). To further test if TNBC cells might be more dependent on glycolysis for cell proliferation and viability, breast cancer cells were cultured in medium containing various concentrations of glucose or with only galactose, which generates ATP mainly through mitochondrial metabolism and can only support the cells with competent mitochondrial function. As shown in Additional file 2: Figure S1D, when TNBC cells were grown in medium containing high glucose (3.15 g/L) they exhibited rapid proliferation and the cell numbers increased 4- to 8-fold by day 4. The cell proliferation rate became slower when glucose was reduced to 1.0 g/L. The cells failed to proliferate and even showed a decrease in cell number when the culture medium contained only galactose (Additional file 2: Figure S1D, F). In contrast, ER-positive cells were able to maintain cell viability and showed moderate proliferation with low-glucose or in galactose-only medium (Additional file 2: Figure S1E, F). Furthermore, the high dependency of TNBC cells on glucose was further demonstrated in clonogenic assays (Additional file 3: Figure S2). TNBC cells exhibited much lower ability to form colonies in low-glucose or galactose medium. Analysis of cellular ATP showed that all subtypes of breast cancer cells had similar ATP content (Figure 1C), suggesting that the upregulation of glycolysis in TNBC cells might be sufficient to compensate for the decreased ATP generation in their mitochondria. These data together further confirm that TNBC cells with low mitochondrial respiration were highly dependent on glucose, and that the presence of high glucose provide the key metabolic substrate for generation of ATP and metabolic intermediates for lipid and nucleic acid synthesis to support the robust growth of TNBC cells.

Because the mitochondrial electron transport chain is a major site of ROS production, we used the mitochondrial redox fluorophore MitoSOX™ Red to measure mitochondrial ROS and found that TNBC cells had higher ROS compared to other breast cancer cells (Additional file 4: Figure S3A, left panel), suggesting an increase in electron leakage from the respiratory chain to form ROS within the mitochondria. The specificity of superoxide detection was confirmed using DHE, and the results were similar with those obtained by MitoSOX™ Red analysis (Additional file 4: Figure S3A, right panel). Consistent with the possible impairment in electron flow through the mitochondrial respiratory chain, the TNBC cells exhibited a significant increase in NADH/NAD + ratio (Figure 1D), likely due to less oxidation of NADH to NAD + by the compromised complex I activity and more conversion of NAD + to NADH during active glycolysis. We have also measured NADPH/NADP + ratio in breast cancer cells, and showed that TNBC cells had a slightly lower NADPH/NADP + ratio compared with the receptor-positive cells (Additional file 4: Figure S3B), which might reflect more consumption of NADPH to counteract higher ROS in TNBC cells. There was also a decrease in cellular glutathione (GSH) level (Additional file 4: Figure S3C) and an increase in glutathione peroxidase 1 expression (GPX-1) (Additional file 4: Figure S3D), reflecting cellular response to increased ROS generation. In addition, TNBC cells exhibited an increase in mitochondria mass (Additional file 4: Figure S3E), suggesting a compensatory response to the impaired respiratory function. The mitochondrial transmembrane potential (Δψm) was higher in TNBC cells as indicated by a higher Rhodamine 123 signal (Additional file 4: Figure S3F), suggesting that the integrity of the mitochondrial inner membranes was maintained.

To further investigate the mitochondrial alterations in TNBC cells, we used oligomycin in conjunction with oxygen consumption analysis to evaluate the mitochondrial OXPHOS coupling efficiency (MCE) [29]. Oligomycin suppresses OXPHOS activity by inhibiting ATP synthase. As shown in Figure 1E, although TNBC cells (MDA-468) exhibited substantially lower basal oxygen consumption compared to the triple-positive breast cancer cells (BT474), the presence of oligomycin (10 ng/ml) caused a similar decrease in oxygen consumption (approximately 50%) in both cell lines, suggesting that TNBC cells had similar mitochondrial coupling efficiency compared to other breast cancer cells. Similar results were observed in other cell lines (Figure 1F), indicating no significant impairment in oxygen consumption/ATP synthesis coupling in TNBC.

We then compared the spare respiratory capacity (SRC) in TNBC cells and other breast cancer cells. SRC was calculated as the difference between the basal mitochondrial O_2_ consumption and the maximal mitochondrial O_2_ consumption when the respiratory chain was uncoupled by CCCP [30]. In the presence of CCCP, the respiratory chain activity is independent from ATP synthesis at complex V (F_1_F_0_ ATPase). Addition of CCCP (4 μM) to BT474 and MCF7 cells resulted in a dramatic increase in mitochondrial respiration (OCR), suggesting that these cells exhibit a high reserve of mitochondrial respiratory capacity (Figure 1E, F). In contrast, TNBC cells seemed to have much lower respiratory reserve capacity, as they exhibited only a limited increase in OCR when CCCP was added (Figure 1E, F). As CCCP acts as a protonophore across all membranes, acidifying the cytosolic compartment and disrupting the function various cellular compartments, this could affect substrate supply and thus affect the uncoupled rate. For this reason CCCP might have different oxidation rates in different breast cancer cells and thus potentially contribute to the differences in the maximum respiratory rates observed to some degree.

### TNBC cells exhibit defect in mitochondrial electron transport complexes activities

**Figure 2:**
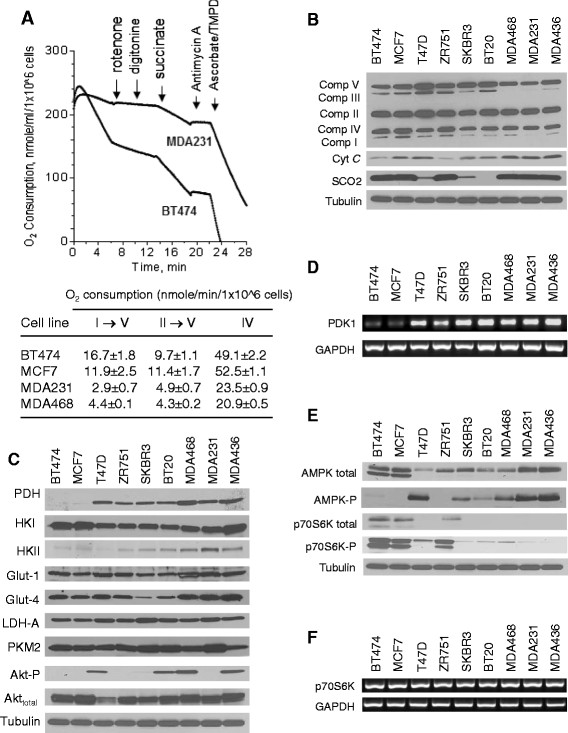
**Analysis of mitochondrial respiratory complex activity and expression of molecules involved in energy metabolism in triple-negative breast cancer (TNBC) cells in comparison with other subtypes of breast cancer cells. (A)** Analysis of mitochondrial respiratory complex activity in breast cancer cells with different receptor status with the Clark electrode using the oxythem system as described in Methods. The representative oxygen consumption curves for BT474 cells (triple-positive) and MDA231 (triple-negative) are shown on the top panel; the quantitative data for each segment of the mitochondrial respiratory chain activity in four different cell lines (two triple-negative lines: MAD231 and MDA468; two receptor-positive lines: BT474 and MCF7) are shown in the lower panel. Data are mean ± SD (n = 3). **(B)** Expression of mitochondrial respiratory chain components in breast cancer cells. The protein levels of complex I subunit NDUFB8 (ND6), complex II 30 kDa subunit, complex III Core 2 subunit, Complex IV subunit II, and the ATP synthase subunit α, cytochrome *c* (cyt c) and SCO2 were determined by western blot analysis. A representative blot from at least three independent experiments is shown. **(C)** Expression of PDH, HKII, HKI, Glut-1, Glut-4, LDH-A, PKM2, total Akt and phospho-Akt (ser 473) in breast cancer cells determined by western blot. Data are representative of three independent experiments. **(D)** Analysis of PDK1 mRNA expression in breast cancer cells by RT-PCR. Data are representative of three independent experiments. **(E)** Analysis of p70S6K and AMPK status in breast cancer cells by western blot. One representative blot from three independent experiments is shown. **(F)** Analysis of p70S6K expression in breast cancer cells by RT-PCR. Data are representative of three independent experiments.

To further examine the mitochondrial dysfunction in TNBC cells, we measured the activity of each respiratory chain complex using specific complex inhibitors in combination with proper complex substrates in digitonin-permeabilized cells [31],[32]. As shown in Figure 2A, respiration of breast cancer cells permeabilized with digitonin (15 μg/ml) was blocked by rotenone (500 nM, complex I inhibitor), restored by addition of succinate (10 mM, complex II substrate), inhibited again by antimycin A (5 μM, complex III inhibitor). The electron flow through complex IV was resumed with addition of electron donors ascorbic acid (2 mM) and TMPD (0.5 mM), which provided electrons directly to cytochrome *c*, thus allowing an estimation of oxidation through complex IV. As shown in the upper panel of Figure 2A, TBNC cells (MDA231) exhibited very low rotenone-sensitive respiration (complex I) and lower succinate-driven respiration (complexes II-IV) compared to the triple-positive breast cancer cells (BT474). Furthermore, addition of ascorbic acid and TMPD to assay the function of complex IV revealed a smaller increased of oxygen consumption in MDA231 cells compared to BT474 cells (Figure 2A, upper panel). Quantitative analysis of data for each segment of the mitochondrial respiratory chain activity in four different cell lines, two triple-negative lines (MAD231 and MDA468) and two receptor-positive lines (BT474 and MCF7), showed that TNBC cells exhibited functional defects in multiple respiratory complexes, since all segments of the respiratory chain activities in TNBC cells were lower than the receptor-positive lines (Figure 2A, lower panel). We further confirmed these observations by analyzing their mitochondrial respiratory chain components by western blotting, which showed a reduction in expression of complex I and complex III proteins (Figure 2B). However, there were no significant changes in the expression of complex II, IV and V components (Figure 2B). Interestingly, the expression of SCO2, a nuclear-encoded complex IV assembly factor, was missing in BT20 (triple-negative), reduced in SKBR3 cells (ER-/PR-/HER2+) and T47D cells (ER+/PR+/HER2-), and unchanged in other cell lines including three TNBC lines (Figure 2B). The expression of cytochrome *c* was slightly increased in TNBC cells (Figure 2B). These results together suggest that TNBC cells exhibit lower OXPHOS due to dysfunction in several mitochondrial respiratory complexes, particularly complexes I and III.

### Alteration of the AMPK-mTOR pathway in TNBC cells

As TNBC cells display low OXPHOS and high glycolysis, we further examined the expression of Akt, Glut-1/-4, HKII, PKM2, LDH-A, and PDH, as these molecules are known to be involved in regulation of glucose metabolism in cancer [13],[33]. Western blot analysis revealed that Akt was activated in the majority of the TNBC cell lines (BT20, MDA468, MDA436), as evidenced by an increase in Akt phosphorylation at ser473 (Figure 2C). However, although MDA231 expressed a similar level of total Akt protein, this TNBC cell line did not show detectable phosphorylated Akt for a yet unknown reason. Thus, it is possible that some of the TNBC cells could be highly glycolytic without Akt activation. The expression of HKII, Glut-1 and Glut-4 was increased in 3 TNBC cell lines (MDA468, MDA231, MDA436), while HKI expression remained unchanged (Figure 2C). Furthermore, TNBC cells also exhibited high expression of PDK1 mRNA (Figure 2D). PDK1 is known to inactivate pyruvate dehydrogenase (PDH) and thus might contribute to a lower utilization of pyruvate by the mitochondria and higher lactate production.

Because AMP-activated protein kinase (AMPK) is a metabolic sensor that responds to alterations in cellular energy levels to maintain energy balance and stimulate glycolysis in response to metabolic stress through direct phosphorylation and activation of 6-phosphofructo-2-kinase [34],[35], we used western blot analysis to examine the expression of AMPK and phospho-AMPK. TNBC cells exhibited an increase in AMPK activity, evident by elevated Thr172 phosphorylation (Figure 2E). The high phospho-AMPK levels in TNBC cells were inversely associated with low expression of p70S6K protein and its phosphorylation at Thr389 (Figure 2E). The mRNA expression of p70S6K in TNBC cells was as high as that in other breast cancer cells (Figure 2F), suggesting that the difference in p70S6K between TNBC cells and receptor-positive breast cancer cells might be due to a difference in post-transcriptional and post-translational regulation. These data are consistent with the role of AMPK in inactivation of p70S6K [36].

### Suppression of the mTOR-p70S6K axis results in suppression of mitochondrial respiration

**Figure 3:**
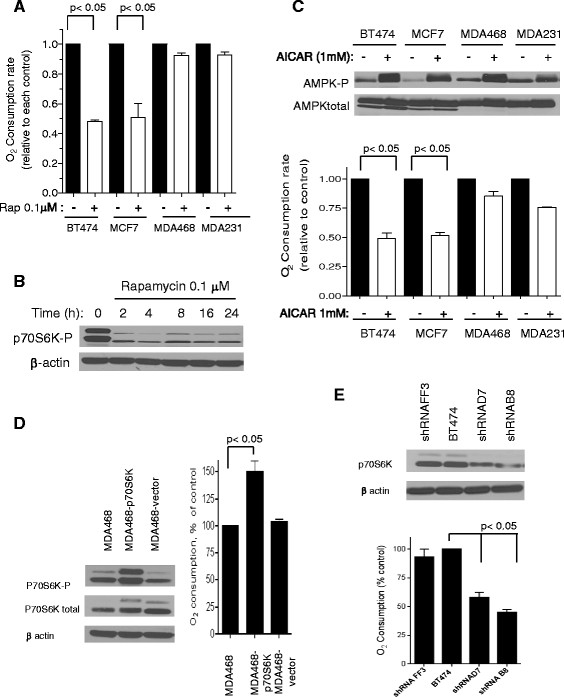
**Modulation of mitochondrial respiration activity in breast cancer cells: mitochondrial respiration is attenuated after inactivation of p70S6K either by rapamycin or p70S6K shRNA and activated after over expression of p70S6K. (A)** Effect of rapamycin on mitochondrial respiration. Breast cancer cells (1x10^6 cells) were treated with 0.1 μM rapamycin for 24 hours, and mitochondrial respiration was measured using the oxytherm system (Clark electrode) in growth medium. **(B)** Inhibition of p70S6K phosphorylation by rapamycin. BT474 cells were treated with 0.1 μM rapamycin for up to 24 hours and cell lysate were analyzed for phospho-p70S6K by western blotting. **(C)** Effect of AMP activator (AICAR) on the mitochondrial respiration of breast cancer cells. Top panel, activation of AMP-activated protein kinase (AMPK) by AICAR. Cells were treated with 1 mM AICAR for 24 hours and cell lysates were probed for AMPK status. Bottom panel, effect of AICAR on mitochondrial respiration. Cells (1 × 10^6 cells) were treated with 1 mM AICAR for 24 hours and mitochondrial respiration was evaluated with the Clark electrode in growth medium. **(D)** Effect of over expression of p70S6K on mitochondrial respiration in MDA468 cells. Left panel, over-expression of P70S6K was first confirmed by western blot. Right panel, mitochondrial respiration of MDA468-p70S6K, vector control, and the parental cell line MDA468 (1 × 10^6 cells) were determined using oxytherm system. **(E)** Knockdown of p70S6K in BT474 decreased the mitochondrial respiration. Top panel, knockdown of p70S6K in BT474 was first confirmed by western blot in several clones. BT474 cells were transfected with control ShRNA (FF3 clones) and p70S6K ShRNA (clones D7 and B8), then clones were selected by controlling the expression of p70S6K. Bottom panel, comparison of the mitochondrial respiration between clones of ShRNA (FF3, B8 and D7) and the parental cell line BT474 (1 × 10^6 cells) measured by Clark electrode. Each bar graph represents mean ± SD from three experiments; *Pp <0.05. One representative from at least three independent experiments is shown for each western blot.

To evaluate the potential role of the altered AMPK-mTOR pathway in affecting mitochondrial function in TNBC cells, we then tested if suppression of this pathway by the mTOR inhibitor rapamycin or the AMPK activator AICAR would lead to changes in mitochondrial respiration. Treatment of the receptor-positive breast cancer cells (BT474 and MCF7) with rapamycin resulted in a rapid inactivation of the mTOR downstream molecule p70S6K, evidenced by a decrease in phospho-p70S6K at Thr389, and a substantial reduction of oxygen consumption, suggesting that the mTOR/p70S6K pathway was important for maintaining mitochondrial respiration in these breast cancer cells (Figure 3A, B). We noted that the mTOR downstream target molecules p70S6K and p85S6K were both detected by the antibody used in immunoblotting (Figure 3B), as p85S6K is derived from the same gene and is identical to p70S6K except for 23 extra residues at the amino terminus [37]. The phosphorylation of p70S6K at threonine 389, however, is known to correlate with S6K activity [38]. Interestingly, the same concentration of rapamycin (0.1 μM) showed no effect on the respiration in TNBC cells (MDA468 and MDA231, Figure 3A), as these cells express very low levels of p70S6K and had low basal respiration. AICAR treatment resulted in activation of AMPK in all breast cancer cells tested, as shown by an increased in the phosphorylation of AMPK at Thr172 (Figure 3C, top panel). However, AICAR only caused a significant decrease of mitochondrial respiration in BT474 and MCF7 cells, while the respiration in TNBC cells (MDA468 and MDA231) was only slightly affected (Figure 3C , bottom panel), consistent with the observation in the experiments using rapamycin.

To further test the role of p70S6K in affecting mitochondrial respiration, we ectopically expressed p70S6K in TNBC cells (MDA468), and showed that such forced expression of p70S6K led to an increased expression of the active protein and caused a significant increase in mitochondrial respiration (Figure 3D). This was associated with a decrease in lactate production and a reduction in glucose uptake (Additional file 5: Figure S4A, B), suggesting that the MDA468 cells with forced p70S6K expression were more active in mitochondrial OXPHOS with less glycolysis. Similarly, reduction of p70S6K expression in triple-positive cells (BT474) by shRNA knockdown led to a significant decrease in respiration (Figure 3E), an increase in glucose uptake and lactate production (Additional file 5: Figure S4C, D). These data together suggest that the AMPK-mTOR-p70S6K axis play a significant role in maintaining mitochondrial respiration in breast cancer cells, and that its loss in TNBC cells led to respiration defect.

### Inhibition of ER, PR, and HER2 leads to activation of AMPK, suppression of p70S6K, and decrease in mitochondrial respiration

**Figure 4:**
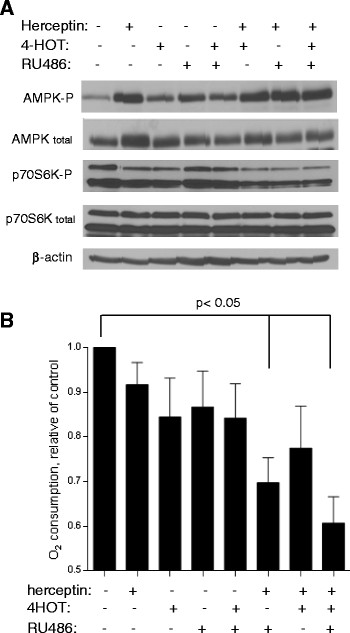
**Effect of herceptin, 4-hydroxy-tamoxifen (4-HOT) and RU486 on the mitochondrial respiration and on p70S6K status in BT474 cells. (A)** Effect of antagonist of breast cancer receptors on AMPK and p70S6K status. BT474 cells were pre-treated with herceptin for 24 hours before adding 4-HOT/or RU486 for an additional 24 hours. Cell lysates were subjected to analysis of p70S6K and AMPK status. Data are representative of at least three independent experiments. **(B)** Mitochondrial respiration was determined using the Clark electrode after addition of the inhibitor as indicated in growth medium. BT474 cells (1 × 10^6 cells) were pre-treated with herceptin for 24 hours before adding 4-HOT/or RU486 for an additional 24 hours (the bar graph represents mean ± SD from three experiments).

As TNBC exhibited significant alteration in AMPK and p70S6K expression and in the mitochondrial respiration, we then investigated if inhibition of ER, PR, and HER2 signaling could modulate the AMPK-p70S6K pathway and affect mitochondrial respiration in breast cancer cells. Herceptin, RU486, and 4-HOT were used to inhibit HER2, PR, and ER, respectively, in BT474 cells (triple-positive). As shown in Figure 4A, inhibition of each receptor with the corresponding compound at sub-toxic concentrations (100 nM 4-HOT, 100 nM RU486, 10 μg/ml herceptin) caused a moderate activation of AMPK and a decrease in phospho-p70S6K, while combinations of these inhibitors resulted in further activation of AMPK and more inhibition of p70S6K, with the combination of all three inhibitors having the strongest effect. Consistently, each inhibitor had a moderate effect on mitochondrial respiration (approximately 15% reduction compare to the control), and the combination of herceptin with either RU486 or 4-HOT caused a further decrease in oxygen consumption (approximately 20 to 30%). Inhibition of all three receptors by combining compounds led to a substantial loss of respiration by 40% (Figure 4B). Under these conditions, we did not observe a significant decrease in cell proliferation (measured by counting cell numbers) or any increase in cell death using annexin-V/PI double staining (data not shown). These data together suggest that suppression of ER, PR, and HER2 signaling in breast cancer cells results in activation of AMPK and suppression of p70S6K, leading to a decrease in mitochondrial respiration.

### TNBC cells are more sensitive to glycolytic inhibition than other subtypes of breast cancer cells

**Figure 5:**
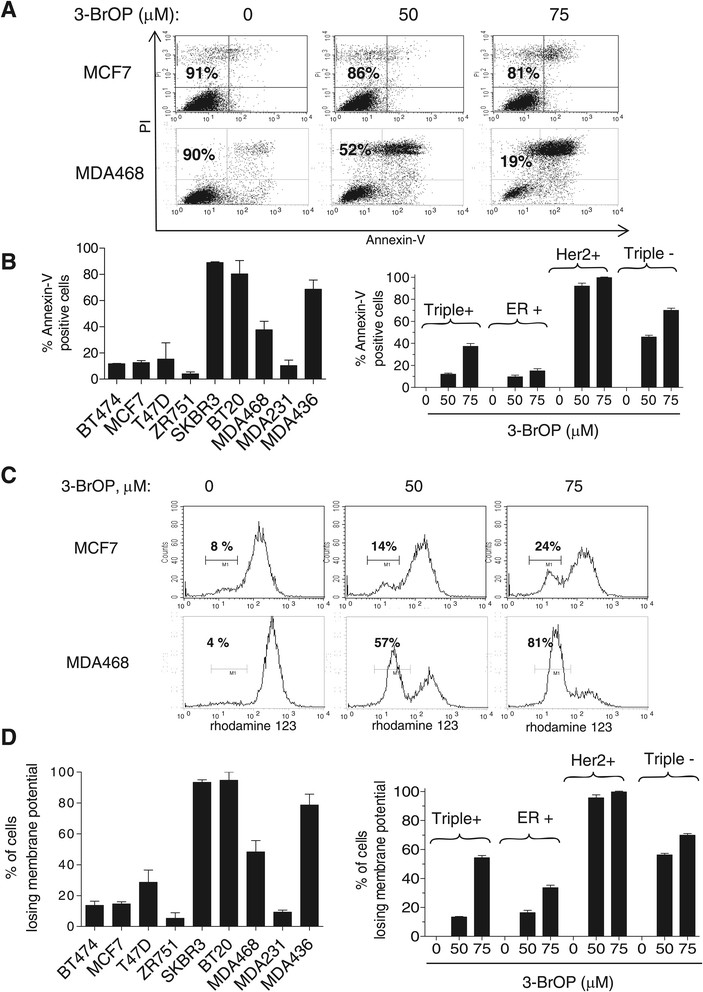
**Breast cancer cells display differential cytotoxic responses to 3-bromo-2-oxopropionate-1-propyl ester (3-BrOP): triple-negative beast cancer (TNBC) cells are more sensitive to 3-BrOP compared to estrogen receptor (ER)-positive cells. (A)** Representative profile of fluorescence-activated cell sorting (FACS) analysis after annexin-V/PI staining. After treatment with 3-BrOP (up to 75 μM) for 24 hours, MDA468 and MCF7 cells were assayed for cell death. Number shown in each panel indicates percentage of the annexin-V and PI double-negative cells (viable). **(B)** Cell death induced by 50 μM 3- BrOP for 24 hours in breast cancer cells detected by flow cytometric analysis (annexin V/PI double staining, left panel). 3-BrOP induced cell death in a dose dependent manner in breast cancer cells (right panel). **(C)** Representative profile of FACS analysis after rhodamine 123 staining. MCF7 and MDA468 were treated with 3-BrOP (up to 75 μM) for 24 hours , the mitochondrial outer membrane potential was then assessed by rhodamine 123 labeling and the reduction of rhodamine 123 fluorescence intensity indicates impaired mitochondrial membrane potential. The number shown in each panel indicates the percentage of cells with mitochondrial potential loss. **(D)** Loss of membrane potential induced by 50 μM 3-BrOP for 24 hours in breast cancer cells detected by flow cytometric analysis (left panel). 3-BrOP induced loss of membrane potential in a dose-dependent manner in breast cancer cells (right panel). The bar graph represents mean ± SD from results of three experiments. Triple+: BT474 cells; ER+: MCF7, T47D, ZR751; Her+: SKBR3, Triple-: BT20, MDA468, MDA231, MDA436.

Based on the observations that TNBC cells exhibited impaired mitochondrial function and have an active glycolytic metabolism, we hypothesized that TNBC cells might be more dependent on glycolysis and thus more sensitive to glycolytic inhibition. Two glycolytic inhibitors, 2-deoxyglucose (2-DG) and 3-bromo-2-oxopropionate-1-propyl ester (3-BrOP), were used to test this possibility. As shown in Figure S5A (Additional file 6), the 4 TNBC cell lines tested were sensitive to growth inhibition by 2-DG, with an average IC50 value of approximately 10 mM. In contrast, the triple positive breast cancer cells (BT474), which have competent mitochondrial respiratory function, were less sensitive to glycolytic inhibition by 2-DG with an IC50 value of approximately 50 mM (Additional file 6: Figure S5B). The ER-positive/HER2-negative cells exhibited intermediate sensitivity (Additional file 6: Figure S5C). Interestingly, SKBR3 cells, which are ER-/PR-/HER2+ with defective mitochondrial respiration (Figure 1), were also more sensitive to 2-DG (Additional file 6: Figure S5D). Consistently, a more potent glycolytic inhibitor 3-BrOP showed a stronger inhibitory effect on the TNBC cells and caused massive cell death in TNBC cells as revealed by annexin-V/PI double-staining (Figure 5A, B) and measurement of mitochondrial transmembrane potential (Figure 5C, D), while the respiration-competent BT474 and MCF7 cells were much less sensitive to 3-BrOP (Figure 5). Most TNBC cells and ER-/PR-/HER2+ cells (SKBR3) cells with low mitochondrial respiration were highly sensitive to 3-BrOP compared to other breast cancer cells with competent mitochondrial respiration (Figure 5B, D). Interestingly, MDA231 cells (TNBC) exhibited only modest response to 3-BrOP for yet unknown reasons.

We further tested if manipulation of P70S6K could have any influence on cell death. As shown in Figure S6 (Additional file 7), the inhibition of p70S6K by rapamycin and by shRNA rendered BT474 cells more sensitive to glycolysis inhibition (Additional file 7: Figure S6A, B). In contrast, over-expression of P70S6K in TNBC cells (MDA468) rendered cells more resistant to 3-BrOP (Additional file 7: Figure S6C). Furthermore, activation of AMPK by AICAR could also make BT474 cells more vulnerable to cell death by 3-BrOP (Additional file 7: Figure S6A). Altogether these data suggest that manipulation of p70S6K/AMPK could sensitize breast cancer cells to the cytotoxicity of glycolytic inhibitors.

**Figure 6:**
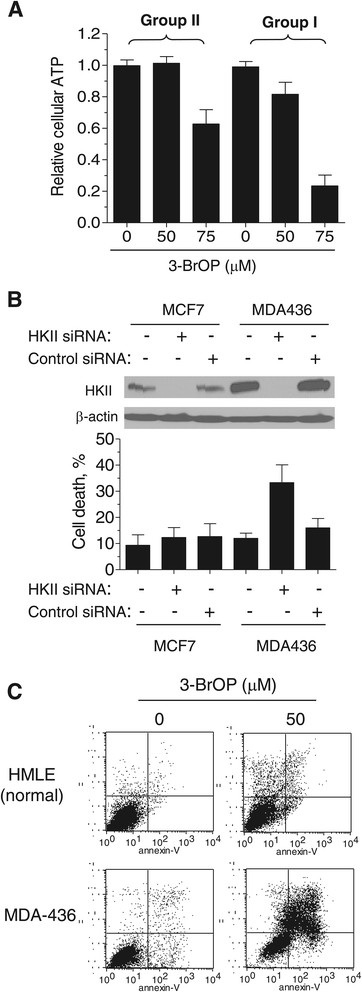
**Triple-negative breast cancer (TNBC) cells are more sensitive to 3-bromo-2-oxopropionate-1-propyl ester (3-BrOP) than other subtypes of breast cancer cells due to its effect on ATP level and their dependence on glycolysis for survival. (A)** 3-BrOP caused more severe depletion of ATP in TNBC and SKBR3 cells (Group I: BT20, MDA468, MDA231, MDA436, SKBR3) compared to estrogen receptor (ER)/progesterone receptor (PR)-positive cells (Group II: BT474, MCF7, T47D, ZR751). Breast cancer cells were treated with 3-BrOP at the indicated concentration for 8 hours. Intracellular ATP level was measured; bar graph represents mean ± SD from three experiments with triplicate wells for each assay. **(B)** TNBC cells are more dependent on glycolysis for survival. Top panel, confirmation of HKII downregulated expression by western blotting in ER-positive cells (MCF7) and TNBC cells (MDA436) after HKII siRNA transfection (72 hours). Data are representative of at least three independent experiments. Bottom panel, TNBC cells are more dependent on HKII for survival assessed by annexin-V/Pi staining. MCF7 and MDA436 cells were transfected with HKII siRNA or scramble siRNA and 72 hours after transfection cell death was detected by flow cytometric analysis using annexin V/PI double staining (bar graph represents mean ± SD from three experiments). **(C)** Representative profile of fluorescence-activated cell sorting analysis after Annexin-V/PI staining. 3-BrOP is more toxic to cancer cells than normal cells. MDA436 cells and immortalized human mammary epithelial cells (HMLE) were treated with 50 μM 3-BrOP (24 h). Cell death was detected by flow cytometric analysis using annexin V/PI double staining.

We then determined the effect of 3-BrOP on ATP levels in breast cancer cells. 3-BrOP induced a more severe loss of ATP in TNBC cells and ER-/PR-/HER2+ (SKBR3) cells (group I) compared to triple-positive and ER-positive cells (Group II, Figure 6A), consistent with the high dependency of TNBC cells on glycolysis for generation of ATP. To further test the dependency of TNBC cells on glucose metabolism in comparison with the mitochondria-competent breast cancer cells, we used siRNA strategy to knock down HKII expression in MDA436 cells (TNBC) and in MCF7 cells (mitochondria-competent), and then examined the effect of such knockdown on cell viability. As shown in Figure 6B, the basal HKII expression level was much higher in MDA436 cells than in MCF7 cells, consistent with the high glycolytic activity in TNBC cells. Importantly, although siRNA was able to completely knockdown the expression of HKII in both cell lines, such a knockdown caused only moderate cell death in MCF7 cells while significantly more apoptotic cells were observed in MDA436 cells (Figure 6B, bottom panel). These results indicate that TNBC cells were more dependent on the glycolysis and that targeting key glycolytic enzyme(s) was effective in killing TNBC cells.

To evaluate the potential therapeutic selectivity of glycolytic inhibition, we compared the sensitivity of TNBC cells to 3-BrOP with immortalized human mammary epithelial cells (HMLE). As shown in Figure 6C, 3-BrOP at 50 μM caused only moderate cytotoxic effect in HMLE, whereas this concentration of 3-BrOP induced massive cell death in MDA436 cells, suggesting that inhibiton of glycolysis might selectively kill breast cancer cells with compromised mitochondrial function.

## Discussion

Glycolysis leads to the production of pyruvate, which can either enter mitochondria for further metabolism through OXPHOS to produce ATP or can be converted to lactate with a simultaneous oxidation of NADH to NAD^+^. Cells with compromised mitochondrial respiration (for example, under hypoxic conditions) switch to higher rates of glycolysis and generate high amounts of lactate. Our study indicates that TNBC cells exhibit impaired mitochondrial function and are more dependent on glycolysis compared to other subtypes of breast cancer cells, even under normoxic conditions. We observed a significantly lower respiratory rate in TNBC cells, associated with decreased expression of complex I and complex III protein components, and an increased expression of glycolytic molecules including Glut1/4 and HKII and activation of Akt that promotes glycolysis. The increase in glucose uptake by TNBC cells (Additional file 2: Figure S1B) will help to provide metabolic intermediaries essential for lipid and nucleic acid syntheses (NADPH and PPP pathway) required for their active proliferation. Indeed, compared with the triple-positive cells, TNBC cells showed higher proliferation rates in the presence of high glucose (Additional file 2: Figure S1D, E). We also showed that TNBC cells were more sensitive to glycolytic inhibition by 2-DG and 3-BrOP compared to ER-positive cells (Figure 5; Additional file 6: Figure S5), due to their lower rates of OXPHOS and greater reliance on glycolysis for production of energy and metabolic intermediates.

The tumor suppressor p53 is known to affect mitochondrial respiratory activity through regulating SCO2 expression [20]; and a loss of p53 function may impair the mitochondrial respiratory chain activity and promote a shift from OXPHOS to glycolysis. In our study, all TNBC cells contained mutated p53. Thus, it is possible that p53 mutation in TNBC might contribute to high glycolysis in these cells. However, among the ER + cell lines, ZR751 and MCF7 have wild-type p53 while T47D and BT474 contain mutant p53 [39], but all four ER + cell lines exhibited similar metabolic profiles manifested by relatively high mitochondrial respiration and low glycolysis. Thus, p53 status alone could not explain the metabolic phenotype observed in breast cancer cells. Our study suggests that the mTOR/p70S6K status may be more closely correlated with high glycolytic activity, while decrease in mTOR/p70S6K activity is correlated with AMPK activation (Figure 2E). Paradoxically, AMPK has been considered a tumor suppressor, while active mTOR has been associated with tumor progression in certain cancers [40]. These differences suggest that the roles of AMPK and mTOR/p70S6K in metabolic regulation and cancer development are highly complex, and may depend on the genetic background of different cells and their stages in the carcinogenesis process. Indeed, the association between intermediary metabolism and tumors may vary over time, with AMPK functioning as a tumor suppressor in pre-malignant cells while the malignant cells undergo a glycolytic switch in part by tolerating AMPK activation [41].

Based on the observations that TNBC cells exhibit activated AMPK, which in turn inhibits mTOR signaling, the altered AMPK/mTOR /p70S6K pathway in TNBC cells may play an important role in suppression of mitochondrial respiration in these cells. We demonstrated that forced expression of p70S6K stimulated mitochondrial respiration while knockdown of p70S6K suppressed mitochondrial respiration (Figure 3). Activated AMPK can suppress mTOR signaling through regulation of TSC2 and raptor [42],[43]. mTOR signaling has also been shown to phosphorylate STAT-3 at residue S727 [44],[45]. STAT3 can interact with GRIM-19, a known component of mitochondrial complex I [46],[47] and regulates mitochondrial respiratory chain [48],[49]. Thus, mTOR–raptor complex may affect mitochondrial activity through several pathways.

Interestingly, normal levels of mitochondrial respiration in receptor-positive cells depend on intact ER, PR and HER2 signaling. We were able to reduce mitochondrial respiration by blocking ER, PR and HER2 signaling in the triple-receptor-positive cells (BT474) using tamoxifen, RU468 and herceptin at low doses that did not induce cell death (Figure 4). We observed an approximately 40% inhibition of mitochondrial oxygen consumption with combined exposure to these inhibitors. The exact signaling link between ER, PR and HER2 receptors and mitochondrial respiration remains unclear and requires further study.

Our study showed that there was a decrease in expression of mitochondrial complexes I and III proteins in TNBC cells. Such a decrease may explain two major biochemical alterations observed in these cells: a decrease in mitochondrial respiration (Figure 1; Additional file 2: Figure S1A) and an increase in mitochondrial ROS generation (Additional file 4: Figure S3A). Mitochondria are major sites of ROS generation, which occurs mainly at complexes I and III of the respiratory chain due to electron leakage from these complexes. Decreased expression of certain chain components might cause discordance in electron transport, leading to an increase in electron leakage and ROS generation. The potential link between increased ROS and elevated mitochondrial transmembrane potential is an interesting area for future study. However, we cannot exclude the possibility that the increased ROS in TNBC cells might also come from other sources such as increased NOX activity [50]. Nevertheless, elevated ROS generation in cancer cells is correlated with the aggressiveness of tumors and poor prognosis [51].

Many cancer cells utilize the glycolytic pathway for energy generation, a phenomenon known as the Warburg effect [3]. In this study, we show that TNBC cells are particularly dependent on glycolysis due to their lower mitochondrial respiration. Our finding is supported by clinical observations. For instance, comparison of PET-CT results between TNBC and other types of breast cancers showed that TNBC exhibit significantly higher ^18^ F-deoxyglucose (FDG) uptake, consistent with elevated glucose utilization [52]. Targeting the key steps in the glycolytic pathway of cancer cells has been shown to produce strong anticancer effects in multiple model systems [8],[53]-[56]. In this study, we found that TNBC cells were more sensitive to glycolytic inhibitors 2-DG and 3-BrOP compared to other sub-types of breast cancer cells. As inhibition of glycolysis creates cellular stress, normal cells may mobilize compensatory mechanisms such as activation of the mTOR pathway that leads to induction of p70S6K phosphorylation and upregulation of nutrient utilization to cope with such a stress [57]. However, TNBC cells have a significant decrease in p70S6K expression and might not be able to effectively counteract glycolytic inhibition. This could be a molecular mechanism that explains their heightened sensitivity to such a metabolic intervention.

Our study showed that the ER/PR−/ER2+ cell line (SKBR3) was metabolically similar to TNBC cells in term of mitochondrial respiration and glycolytic activity. The reason that SKBR3 cells showed low mitochondrial respiration and high glycolysis was likely due to their very low expression of p70S6K and phospho-p70S6K. As downregulation of the mTOR/p70S6K pathway is a key molecular event leading to low mitochondrial respiration, it is not surprising that SKBR3 cells exhibit a glucose metabolic profile similar to TNBC cells, which all have low p70S6K expression. It is important to note that clinically, TNBC cells are not sensitive to receptor-targeted drugs due to the lack of receptor expression, and glycolytic inhibition may provide a potentially effective treatment option. In contrast, ER/PR−/HER2+ breast cancer cells (such as SKBR3) remain sensitive to herceptin and inhibition of glycolysis may provide an additional option for effective treatment.

The PI3K/Akt/mTOR pathway is a complex network with multiple components, and is frequently deregulated in human breast cancer [58],[59]. Based on this rationale, multiple inhibitors against the PI3K/Akt/mTOR axis have been developed. Preclinical studies have shown that breast cancer cell lines with alterations in the PI3K/Akt/mTOR pathway, such as activating *PIK3CA* mutations or HER2 amplification, are sensitive to PI3K/Akt/mTOR pathway inhibition [24],[26],[60]. Recent data indicate that combining endocrine therapy in breast cancer with mTOR inhibitors is effective to eliminate cancer cells [61],[62]. Interestingly, PI3K/AKT/mTOR and ER have been shown to be implicated in bidirectional crosstalk, in which intracellular signaling pathways stimulate genomic ER signaling through phosphorylation and activation of the receptor and its cofactors. In addition, estrogen stimulation of breast cancer cells immediately upregulates intracellular kinase signaling, suggesting nongenomic signaling through cytoplasmic or membrane-bound ER to be involved in activation of PI3K/AKT/mTOR signaling [62]. Basal-like TNBC cells were highly sensitive to everolimus (RAD001) while the other subgroup of TNBC cells comprising cell lines characterized as stem-like cancer cells were less sensitive to this drug [63]. Thus, other therapeutic strategies against these TNBCs are needed and targeting their metabolism might be an attractive option.

## Conclusions

In summary, we have demonstrated that TNBC exhibited a metabolic profile characterized by low mitochondrial oxidative phosphorylation and increased glycolysis compared to other breast cancer cells. This phenotype may be due in part to the constitutive activation of AMPK and low expression of p70S6K. These metabolic alterations may provide a biochemical basis for developing new therapeutic strategies to effectively kill TBNC by glycolytic inhibition. Although three of the four TNBC cell lines used in this study (BT20, MDA468, MDA436) were sensitive to glycolytic inhibition, one TNBC cell line (MDA231) cells exhibited resistance to such metabolic inhibition for a yet unknown mechanism. It is possible that this particular cell line might have acquired drug resistant mechanisms such as over-expression of anti-apoptotic molecule(s) or a defect in cell death pathway, leading to insensitivity to many anticancer agents. Another exception is that one ER/PR-positive cell line (T47D) showed relatively low p70S6K expression but exhibited competent mitochondrial respiration, suggesting that T47D cells might have a mechanism to maintain mitochondrial respiration without high expression of p70S6K. Thus, it is important to consider such exceptions when using glycolytic inhibition as a new strategy to kill breast cancer cells.

## Authors’ contributions

HP designed and performed research, analyzed data and drafted the manuscript; WZ, JL, NH performed research and analyzed data; JD, RHX, LP participated in the design of the study, gave technical assistance and helpful discussions, and critically revised the manuscript for important intellectual content; PH directed the study design, data analysis/interpretation and drafted the manuscript. All authors read and approved the final manuscript for submission and agree to be accountable for all aspects of the work.

## Additional files

## Electronic supplementary material

Additional file 1: Tables S1 to S12.: containing the quantification of Western blots and RNA gels. (PDF 67 KB)

Additional file 2: Figure S1.: is a figure showing that TNBC cells are more dependent on glycolysis than other ER-positive cells. (PDF 63 KB)

Additional file 3: Figure S2.: is a figure showing the impact of glucose and galactose on breast cancer proliferation. (PDF 795 KB)

Additional file 4: Figure S3.: is a figure showing few characterizations of TNBC cells. (PDF 39 KB)

Additional file 5: Figure S4.: is a figure showing the effect of p70S6K on lactate production and glucose uptake in breast cancer cells. (PDF 19 KB)

Additional file 6: Figure S5.: is a figure showing the effect of 2-DG on proliferation of breast cancer cells. (PDF 21 KB)

Additional file 7: Figure S6.: is a figure showing how to sensitize breast cancer cells to glycolysis inhibition. (PDF 20 KB)

Below are the links to the authors’ original submitted files for images.

Authors’ original file for figure 1

Authors’ original file for figure 2

Authors’ original file for figure 3

Authors’ original file for figure 4

Authors’ original file for figure 5

Authors’ original file for figure 6

## Abbreviations

2-DG: 2-deoxyglucose
3-BrOP: 3-bromo-2-oxopropionate-1-propyl ester
4-HOT: 4-hydroxy-tamoxifen
AICAR: AMP activator
ALD: aldolase
AMPK: AMP-activated protein kinase
CCCP: cyanide 3-chlorophenylhydrazone
DHE: dihydroethidium
DMEM: Dulbecco’s modified Eagle’s medium
ECAR: acidification rate
ER: estrogen receptors
GPX-1: glutathione peroxidase 1
GSH: glutathione level
HER2: human epidermal growth factor receptor-2
HK: hexokinase
LDH: lactate dehydrogenase
MCE: mitochondrial coupling efficiency
mTOR: mammalian target of rapamycin
OCR: oxygen consumption rate
OXPHOS: oxidative phosphorylation
PR: progesterone receptors
PTEN: phosphatase and tensin homolog
ROS: reactive oxygen species
SRC: spare respiratory capacity spare respiratory capacity
TMPD: N,N,N’,N’-tetramethyl-p-phenylenediamine dihydrochloride
TNBC: triple-negative breast cancer
Δψm: mitochondrial transmembrane potential
