# Supplementary material for: Mitochondrial dysfunction in some triple-negative breast cancer cell lines: role of mTOR pathway and therapeutic potential
Source: Breast Cancer Res. 2014 Sep 11;16:434. doi: 10.1186/s13058-014-0434-6 (PMC4303115; doi:10.1186/s13058-014-0434-6)
Supplement: Supplementary file 1 — Additional file 1: Tables S1 to S12.: containing the quantification of Western blots and RNA gels. (PDF 67 KB) [file 13058_2014_434_MOESM1_ESM.pdf]

## Table 1: Quantification Figure 2B normalized by its own tubulin

| | BT474 | MCF7 | TD47 | ZR751 | SKB3 | BT20 | MDA468 | MDA231 | MDA436 |
|---|---|---|---|---|---|---|---|---|---|
| complex V | 0.84 | 0.92 | 1.04 | 0.96 | 0.93 | 0.93 | 0.81 | 0.75 | 0.84 |
| complex III | 0.70 | 0.75 | 0.83 | 0.78 | 0.65 | 0.70 | 0.36 | 0.36 | 0.48 |
| complex II | 1.01 | 1.02 | 1.08 | 1.03 | 0.90 | 0.98 | 0.94 | 0.91 | 0.91 |
| complex IV | 0.90 | 0.93 | 0.85 | 0.90 | 0.77 | 0.80 | 0.78 | 0.75 | 0.77 |
| complex I | 0.66 | 0.72 | 0.67 | 0.68 | 0.58 | 0.59 | 0.58 | 0.44 | 0.57 |
| cytochrome *C* | 0.61 | 0.70 | 0.75 | 0.58 | 0.65 | 0.66 | 0.74 | 0.74 | 0.78 |
| SCO2 | 0.87 | 0.90 | 0.62 | 0.89 | 0.57 | 0.27 | 0.91 | 0.91 | 0.90 |

## Table 2: Quantification Figure 2C normalized by its own tubulin

| | BT474 | MCF7 | TD47 | ZR751 | SKB3 | BT20 | MDA468 | MDA231 | MDA436 |
|---|---|---|---|---|---|---|---|---|---|
| PDH | 0.23 | 0.22 | 0.83 | 0.76 | 0.76 | 0.78 | 1.02 | 0.86 | 0.81 |
| HDK1 | 0.87 | 0.89 | 0.77 | 0.73 | 0.72 | 0.71 | 0.78 | 0.82 | 0.96 |
| HDKII | 0.38 | 0.43 | 0.35 | 0.40 | 0.44 | 0.54 | 0.71 | 0.81 | 0.72 |
| Glut1 | 0.82 | 0.79 | 0.90 | 0.85 | 0.74 | 0.77 | 0.96 | 0.93 | 0.87 |
| Glut4 | 0.94 | 0.85 | 0.94 | 0.85 | 0.61 | 0.74 | 1.01 | 1.04 | 1.13 |
| LDH-A | 0.78 | 0.78 | 0.89 | 0.85 | 0.92 | 0.85 | 0.82 | 0.87 | 0.77 |
| PKM2 | 0.89 | 0.89 | 0.87 | 0.91 | 0.94 | 1.02 | 0.96 | 1.07 | 0.82 |
| AKT-P | 0.36 | 0.36 | 0.91 | 0.35 | 0.36 | 0.96 | 1.13 | 0.35 | 1.00 |
| AKT-t | 0.98 | 1.05 | 0.82 | 0.94 | 0.90 | 0.96 | 1.01 | 0.97 | 1.03 |

# Table 3: Quantification Figure 2D normalized by its own GAPDH

| | BT474 | MCF7 | TD47 | ZR751 | SKB3 | BT20 | MDA468 | MDA231 | MDA436 |
|---|---|---|---|---|---|---|---|---|---|
| PDK1 | 0.41 | 0.38 | 1.22 | 1.14 | 1.33 | 1.67 | 1.48 | 1.49 | 1.58 |

# Table 4: Quantification Figure 2E normalized by its own tubulin

| | BT474 | MCF7 | TD47 | ZR751 | SKB3 | BT20 | MDA468 | MDA231 | MDA436 |
|---|---|---|---|---|---|---|---|---|---|
| AMPK total | 0.74 | 0.83 | 0.52 | 0.71 | 0.76 | 0.60 | 0.71 | 0.89 | 0.93 |
| AMPK-P | 0.26 | 0.23 | 1.14 | 0.23 | 0.80 | 0.65 | 0.93 | 1.10 | 1.16 |
| P70S6K total | 0.65 | 0.57 | 0.40 | 0.49 | 0.37 | 0.27 | 0.28 | 0.27 | 0.27 |
| P70S6K-P | 0.86 | 0.72 | 0.47 | 0.70 | 0.25 | 0.27 | 0.31 | 0.24 | 0.23 |

# Table 5: Quantification Figure 2F normalized by its own GAPDH

| | BT474 | MCF7 | TD47 | ZR751 | SKB3 | BT20 | MDA468 | MDA231 | MDA436 |
|---|---|---|---|---|---|---|---|---|---|
| P70S6K | 0.97 | 0.95 | 0.92 | 0.92 | 0.95 | 0.99 | 0.94 | 0.95 | 0.91 |

## Table 6: Quantification Figure 3B normalized by its own β-actin

| time,h | 0 | 2 | 4 | 8 | 16 | 24 |
|---|---|---|---|---|---|---|
| p70S6K-P | 1.01 | 0.39 | 0.35 | 0.31 | 0.34 | 0.38 |

## Table 7: Quantification Figure 3C normalized by its own AMPK total

| | BT474 | | MCF7 | | MDA468 | | MDA231 | |
|---|---|---|---|---|---|---|---|---|
| AICAR | 0.00 | 1mM | 0.00 | 1mM | 0.00 | 1mM | 0.00 | 1mM |
| | 0.66 | 1.05 | 0.55 | 1.02 | 0.74 | 1.05 | 0.74 | 0.92 |

## Table 8: Quantification Figure 3D normalized by its own β-actin

| | MDA468 | MDA468-p70S6K | MDA468-vector |
|---|---|---|---|
| P70S6K-P | 1.3 | 1.42 | 1 |
| P70S6K-total | 0.61 | 0.69 | 0.72 |

## Table 9: Quantification Figure 3E normalized by its own β-actin

| | shRNAFF3 | BT474 | shRNAD7 | shRNAB8 |
|---|---|---|---|---|
| p70S6K | 0.92 | 0.90 | 0.70 | 0.65 |

## Table 10: Quantification Figure 4A normalized by its own β-actin

| | | | | | | | | |
|---|---|---|---|---|---|---|---|---|
| Herceptin | - | + | - | - | - | + | + | + |
| 4-HOT | - | - | + | - | + | + | - | + |
| RU486 | - | - | - | + | + | - | + | + |
| AMPK-P | 0.89 | 1.52 | 1.11 | 1.11 | 1.01 | 1.38 | 1.53 | 1.50 |
| AMPK total | 1.29 | 1.62 | 1.37 | 1.16 | 1.08 | 1.33 | 1.23 | 1.26 |
| p70S6K-P | 1.32 | 1.28 | 1.18 | 1.17 | 1.07 | 1.08 | 1.07 | 1.06 |
| p70S6K total | 1.24 | 1.29 | 1.24 | 1.12 | 1.08 | 1.20 | 1.22 | 1.25 |

## Table 11: Quantification Figure 6B normalized by its own β-actin

| | MCF7 | | | MDA436 | | |
|---|---|---|---|---|---|---|
| | control | HKII siRNA | control shRNA | control | HKII siRNA | control shRNA |
| HKII | 0.98 | 0.15 | 0.94 | 1.37 | 0.17 | 1.28 |

## Table 12: Quantification Figure S3D normalized by its own β-actin

| | BT474 | BT20 | MDA468 | MDA231 | MDA436 |
|---|---|---|---|---|---|
| GPX1 | 0.51 | 0.80 | 0.80 | 0.94 | 0.89 |
