# Supplementary material for: Mitochondrial dysfunction in some triple-negative breast cancer cell lines: role of mTOR pathway and therapeutic potential
Source: Breast Cancer Res. 2014 Sep 11;16:434. doi: 10.1186/s13058-014-0434-6 (PMC4303115; doi:10.1186/s13058-014-0434-6)
Supplement: Supplementary file 2 — Additional file 2: Figure S1.: is a figure showing that TNBC cells are more dependent on glycolysis than other ER-positive cells. (PDF 63 KB) [file 13058_2014_434_MOESM2_ESM.pdf]

Additional File 1

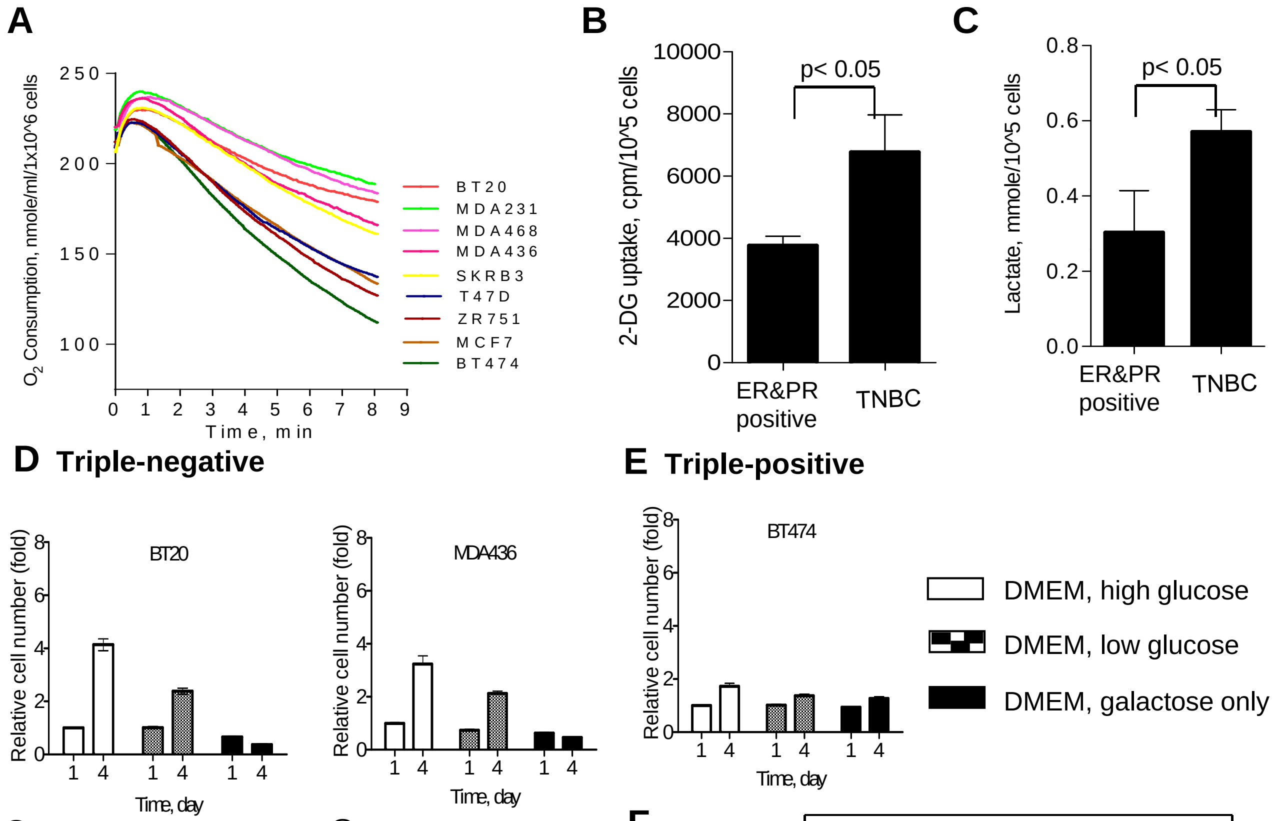

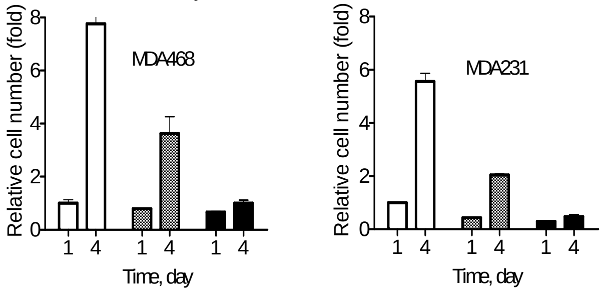

F

medium / cell	Doubling time (h)		
	DMEM , high glucose	DMEM, low glucose	DMEM, galactose
BT474	52.7	56.8	54.5
MCF7	27.6	29.5	32.05
MDA231	27.1	26	38.7
MDA436	26.1	26.7	35.6
MDA468	27.5	31.8	44.1

Figure S1. **TNBC cells are more dependent on glycolysis** . (A) Comparison of oxygen consumption by breast cancer cell lines (1x10^6 cells) using clarck electrode. One representative from at least three independent experiments is shown. (B) Glucose uptake by breast cancer cell lines. Comparison between the TNBC group (BT20, MDA468, MDA231, MDA436) and the ER/PR-positive group (BT474, MCF7, T47D, ZR751). The bar graph represents mean ± SD from three experiments. (C) Lactate production by breast cancer cell lines. Comparison between the TNBC group (BT20, MDA468, MDA231, MDA436) and the ER/PR-positive group (BT474, MCF7, T47D, ZR751). The bar graph represents mean ± SD from three experiments. (D-E), TNBC cells are more dependent on glucose for proliferation and viability. Breast cancer cells triple-negative (D) and triple-positive (E) were cultured in DMEM media containing galactose (3.15g/L) without glucose, DMEM with low glucose (1g/L), or DMEM with normal glucose (3.15 g/L) as indicated. Cell counts were measured on day 1 and day 4. The cell numbers on day 4 are normalized by the number on day 1 with medium containing normal glucose and expressed as relative number (fold). The bar graph represents mean ± SD from 3 experiments. (F) Doubling time of TNBC group (MDA468, MDA231, MDA436) and the ER/PR-positive group (BT474, MCF7).
