# Supplementary material for: Mitochondrial dysfunction in some triple-negative breast cancer cell lines: role of mTOR pathway and therapeutic potential
Source: Breast Cancer Res. 2014 Sep 11;16:434. doi: 10.1186/s13058-014-0434-6 (PMC4303115; doi:10.1186/s13058-014-0434-6)
Supplement: Supplementary file 3 — Additional file 3: Figure S2.: is a figure showing the impact of glucose and galactose on breast cancer proliferation. (PDF 795 KB) [file 13058_2014_434_MOESM3_ESM.pdf]

Additional File 2

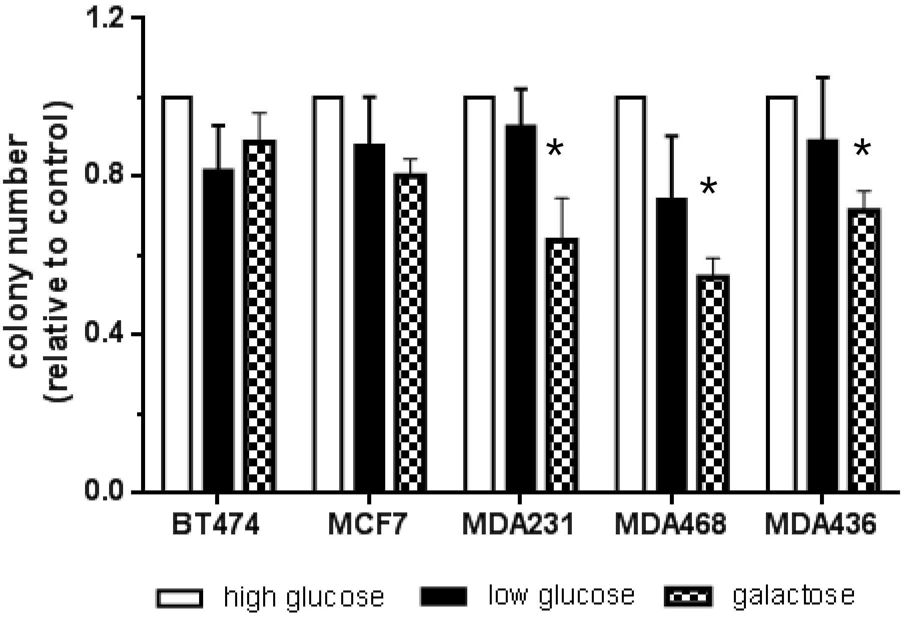

Figure S2. **Effect of sugar on TNBC cell proliferation.** Breast cancer cells (3000 cells) were plated in soft agar with DMEM containing high glucose (3.15 g/L) or low glucose (1g/L), or in DMEM media containing galactose (3.15g/L) without glucose. After 4 weeks, cell colonies were stained and counted. Each data point represents the mean ± S.D. (n = 3). *, $p<0.05$
