# Supplementary material for: Mitochondrial dysfunction in some triple-negative breast cancer cell lines: role of mTOR pathway and therapeutic potential
Source: Breast Cancer Res. 2014 Sep 11;16:434. doi: 10.1186/s13058-014-0434-6 (PMC4303115; doi:10.1186/s13058-014-0434-6)
Supplement: Supplementary file 4 — Additional file 4: Figure S3.: is a figure showing few characterizations of TNBC cells. (PDF 39 KB) [file 13058_2014_434_MOESM4_ESM.pdf]

Additional File 3

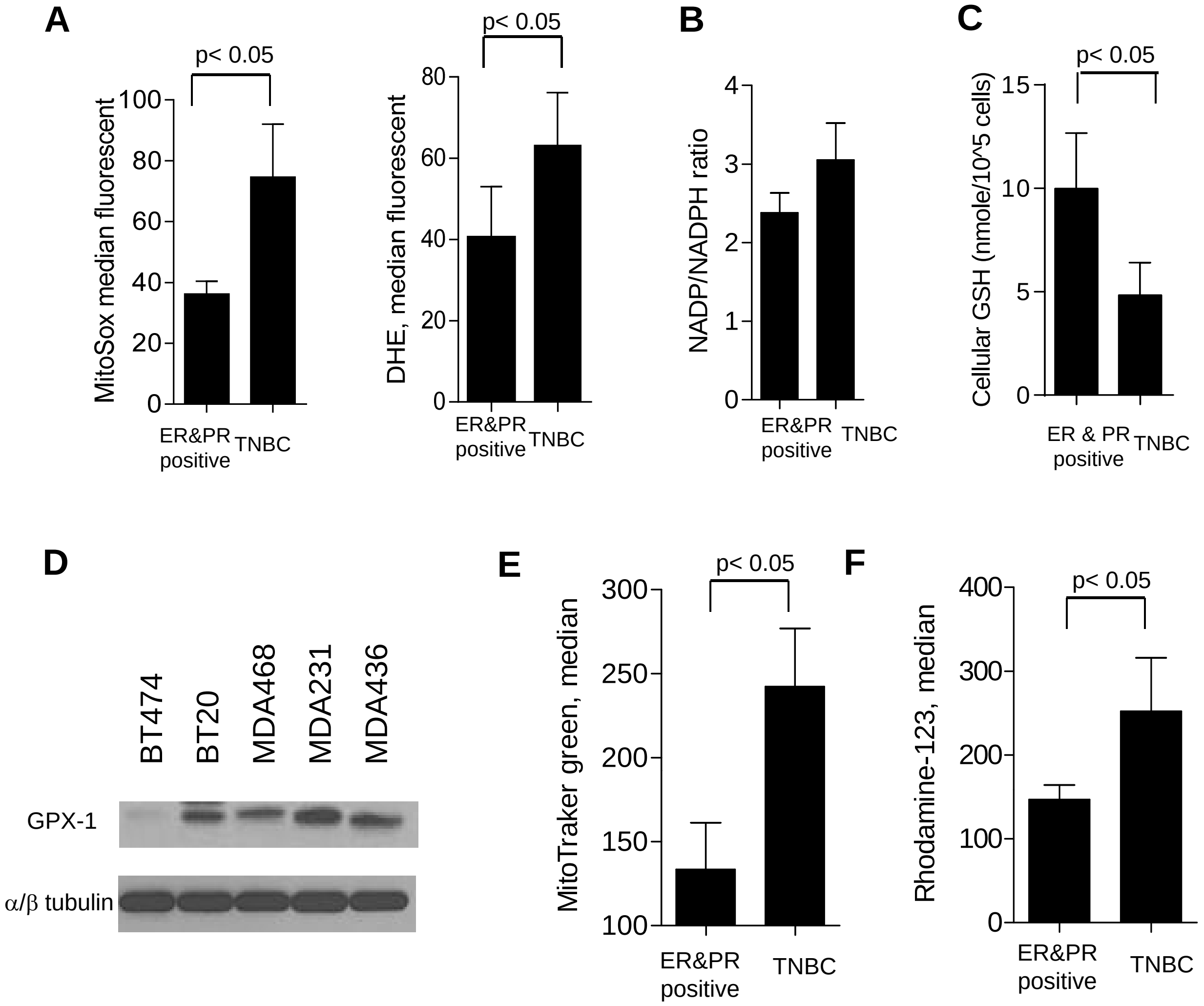

Figure S3. **Characterization of TNBC cells.** *(*A) Levels of ROS in breast cancer cells assessed by MitoSOX^{TM} Red (Mitosox, left panel) and by dihydroethidium (DHE, right panel). (B) Comparison of NADP+/NADPH ratios in breast cancer cell lines between the TNBC group and the ER/PR-positive group. (C) GSH level is lower in TNBC cells compared to ER-PR positive cells. (D) Overexpression of GPX-1 in TBNC cells determined by western blot compare to ER-PR positive cells (BT474). One representative from at least three independent experiments is shown. . (E) TNBC cells exhibit a higher mitochondrial mass compare to ER-PR positive cells. (F) TNBC cells exhibit a higher mitochondrial membrane potential compare to ER-PR positive cells. The bar graph represents mean ± SD from three experiments. Note: TNBC cell lines included BT20, MDA468, MDA231, MDA436; ER/PR-positive cell lines included BT474, MCF7, T47D, ZR751.
