# Supplementary material for: Mitochondrial dysfunction in some triple-negative breast cancer cell lines: role of mTOR pathway and therapeutic potential
Source: Breast Cancer Res. 2014 Sep 11;16:434. doi: 10.1186/s13058-014-0434-6 (PMC4303115; doi:10.1186/s13058-014-0434-6)
Supplement: Supplementary file 5 — Additional file 5: Figure S4.: is a figure showing the effect of p70S6K on lactate production and glucose uptake in breast cancer cells. (PDF 19 KB) [file 13058_2014_434_MOESM5_ESM.pdf]

Additional File 4

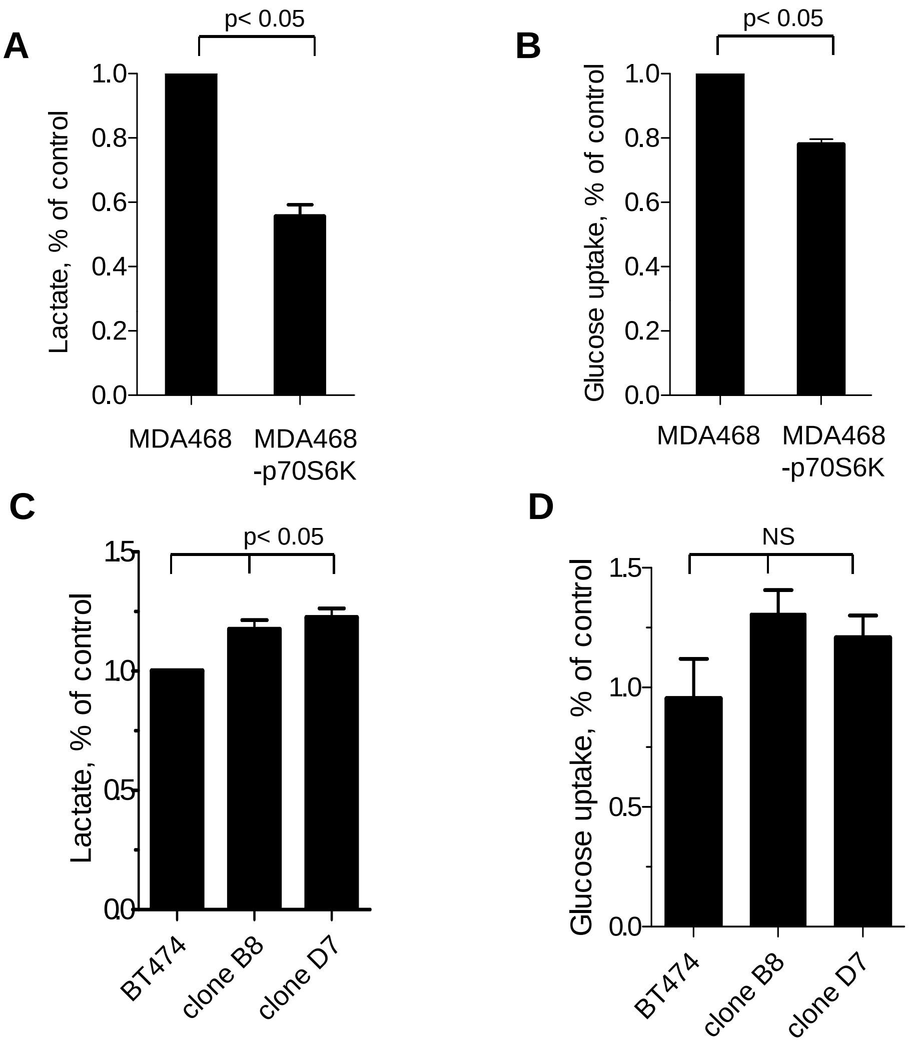

***Figure S4*. Effect of p70S6K on lactate production and glucose uptake.** A-B, MDA468-p70S6K cells exhibit a decrease in glycolysis compared to MDA468 parental cells. (A) Lactate production over 24h is lower in MDA468-p70S6K cells compared to parental MDA468 cells. (B) MDA468-p70S6K uptake less glucose compare to parental MDA468 cells. The bar graph represents mean ± SD from three experiments. (C-D) BT474 shRNA-p70S6K cells exhibit an increase in glycolysis compared to BT474 parental cells. (C) Lactate production over 24h is higher in BT474-p70S6K shRNA cells (B8 and D7 clones) compared to parental BT474 cells. (D) BT474-p70S6K shRNA cells (B8 and D7 clones) uptake more glucose than BT474 cells. The bar graph represents mean ± SD from three experiments.
