# Supplementary material for: Mitochondrial dysfunction in some triple-negative breast cancer cell lines: role of mTOR pathway and therapeutic potential
Source: Breast Cancer Res. 2014 Sep 11;16:434. doi: 10.1186/s13058-014-0434-6 (PMC4303115; doi:10.1186/s13058-014-0434-6)
Supplement: Supplementary file 6 — Additional file 6: Figure S5.: is a figure showing the effect of 2-DG on proliferation of breast cancer cells. (PDF 21 KB) [file 13058_2014_434_MOESM6_ESM.pdf]

Additional File 5

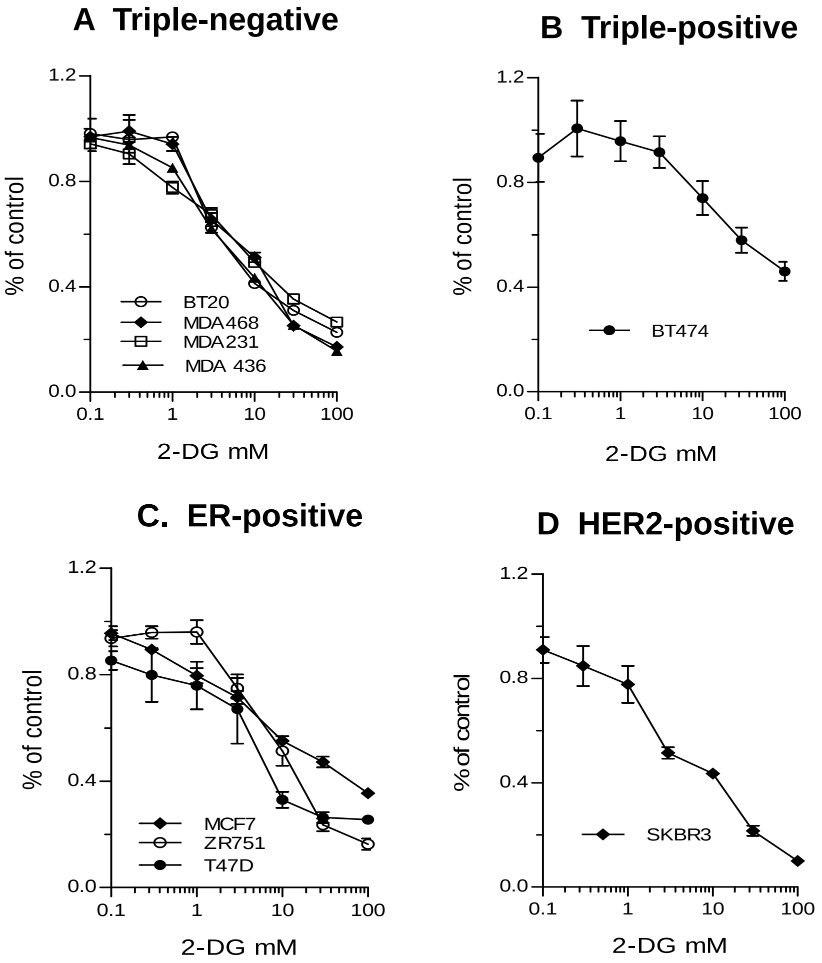

Figure S5. **Effect of 2-DG on proliferation of breast cancer cells with various receptor status.** Breast cancer cells that are triple-negative (A), triple-positive (B), ER-positive (C), and HER-2-positive (D) were incubated with the indicated concentrations of 2-deoxyglucose (2-DG) for 72 hours, and inhibition of cell proliferation was measured using MTS assay (the bar graph represents mean ± SD from three experiments).
