# Supplementary material for: Mitochondrial dysfunction in some triple-negative breast cancer cell lines: role of mTOR pathway and therapeutic potential
Source: Breast Cancer Res. 2014 Sep 11;16:434. doi: 10.1186/s13058-014-0434-6 (PMC4303115; doi:10.1186/s13058-014-0434-6)
Supplement: Supplementary file 7 — Additional file 7: Figure S6.: is a figure showing how to sensitize breast cancer cells to glycolysis inhibition. (PDF 20 KB) [file 13058_2014_434_MOESM7_ESM.pdf]

Additional File 6

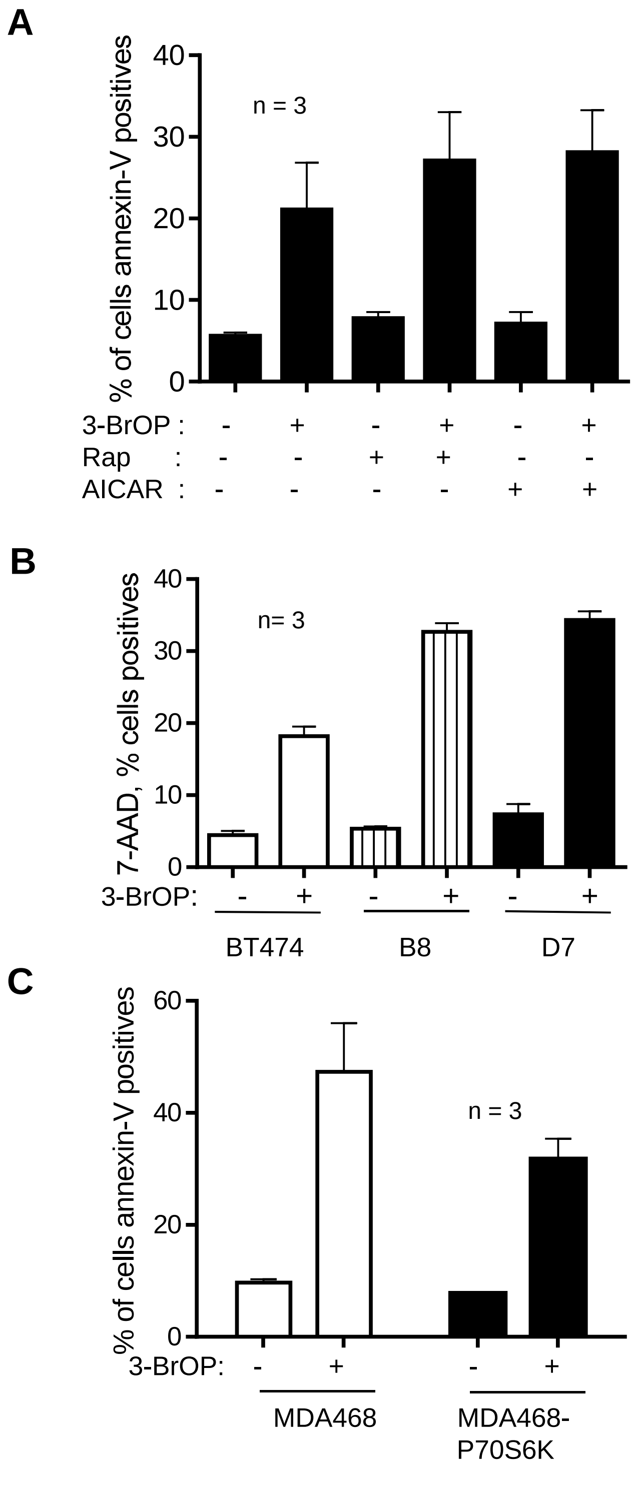

Figure S6. **Sensitization of breast cancer cells to glycolysis inhibition.** (A) Inhibition of mTOR and activation of AMPK render BT474 cells more sensitive to glycolysis inhibition. (B) BT474 cells become more sensitive to 3-BrOP after P70S6K shRNA knockdown. (C) Over-expression of P70S6K in MDA468 (TNBC) cells render cells less sensitive to glycolysis inhibition by 3-BrOP .
