# Supplementary figures and images for: Mitochondrial dysfunction in some triple-negative breast cancer cell lines: role of mTOR pathway and therapeutic potential
Source: Breast Cancer Res. 2014 Sep 11;16:434. doi: 10.1186/s13058-014-0434-6 (PMC4303115; doi:10.1186/s13058-014-0434-6)

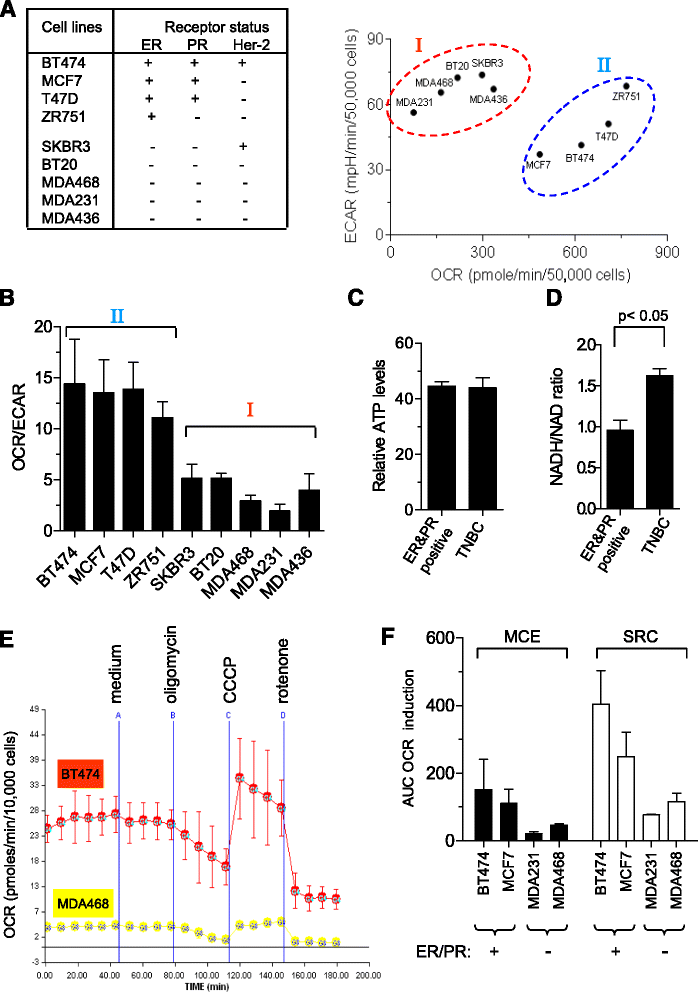

Supplement: Supplementary file 8 — Authors’ original file for figure 1 [file 13058_2014_434_MOESM8_ESM.gif]

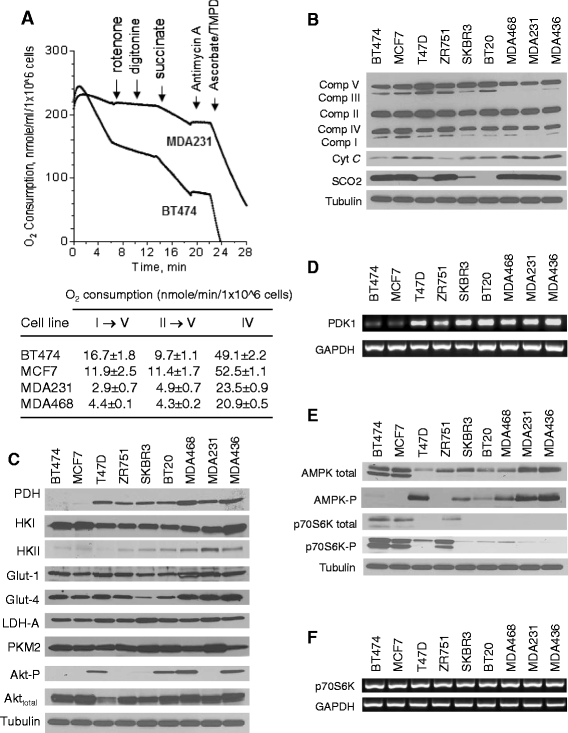

Supplement: Supplementary file 9 — Authors’ original file for figure 2 [file 13058_2014_434_MOESM9_ESM.gif]

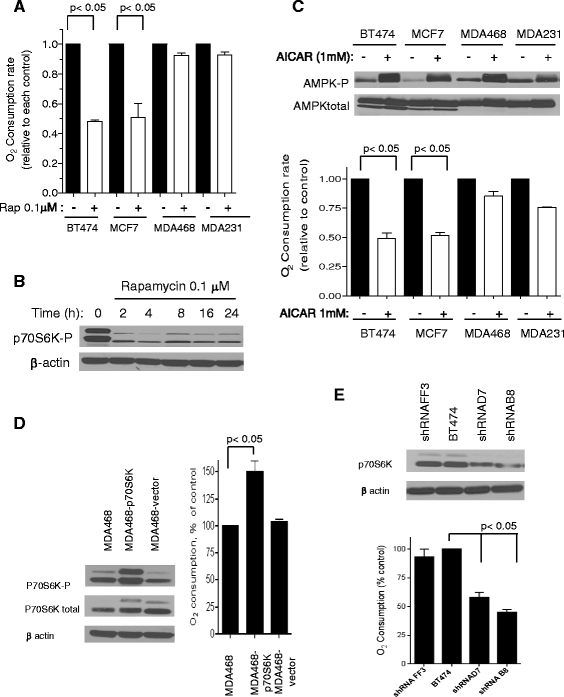

Supplement: Supplementary file 10 — Authors’ original file for figure 3 [file 13058_2014_434_MOESM10_ESM.gif]

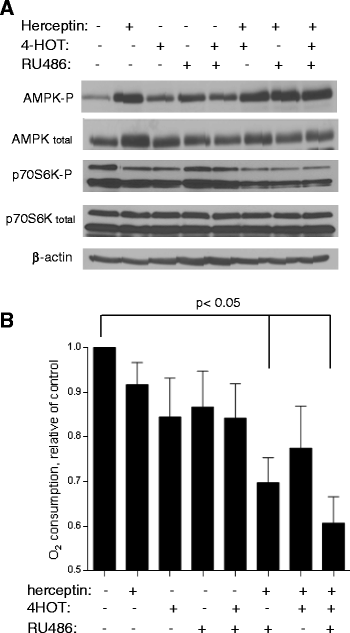

Supplement: Supplementary file 11 — Authors’ original file for figure 4 [file 13058_2014_434_MOESM11_ESM.gif]

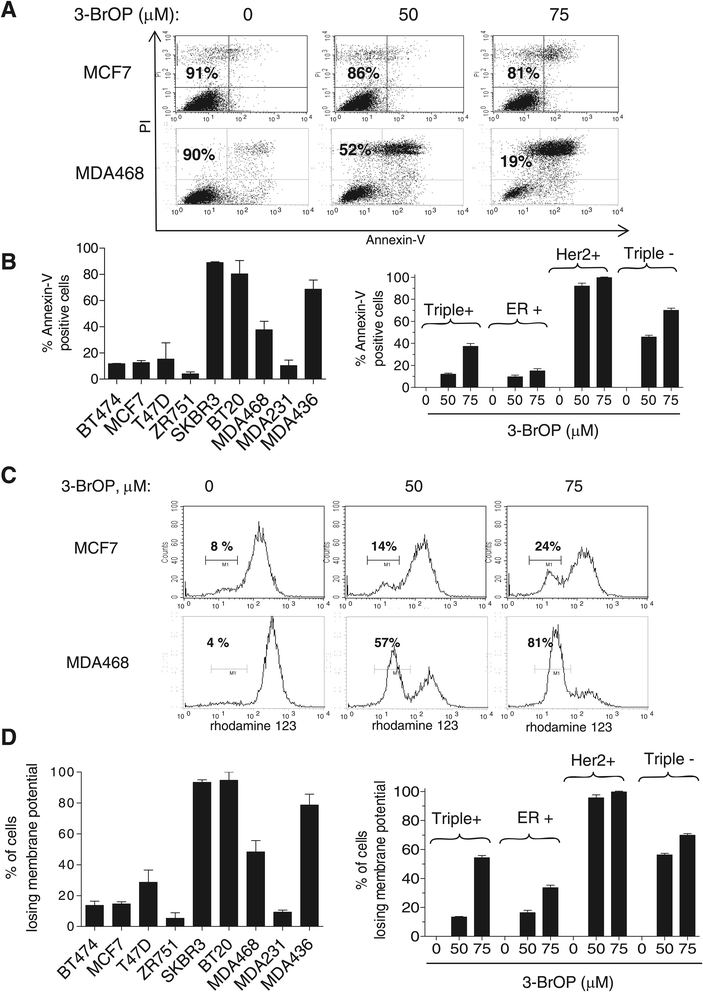

Supplement: Supplementary file 12 — Authors’ original file for figure 5 [file 13058_2014_434_MOESM12_ESM.gif]

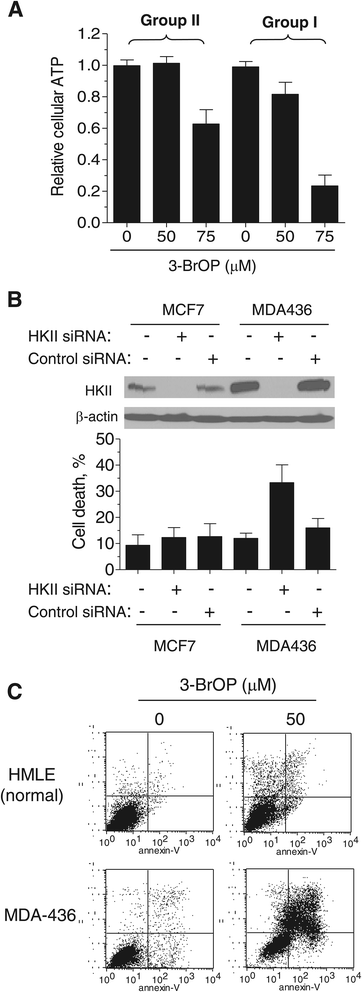

Supplement: Supplementary file 13 — Authors’ original file for figure 6 [file 13058_2014_434_MOESM13_ESM.gif]
